# Correction: The complex dynamics of products and its asymptotic properties

**DOI:** 10.1371/journal.pone.0186436

**Published:** 2017-10-11

**Authors:** Orazio Angelini, Matthieu Cristelli, Andrea Zaccaria, Luciano Pietronero

S19 File is omitted from the list of Supporting Information. It can be viewed below.

## Supporting information

S19 File Additional supporting information. Here we report and discuss a number of auxiliary results supporting the main findings of the principal paper and assess the robustness of our calculations.
(PDF)
