# Supplementary figures and images for: The complex dynamics of products and its asymptotic properties
Source: PLoS One. 2017 May 17;12(5):e0177360. doi: 10.1371/journal.pone.0177360 (PMC5435184; doi:10.1371/journal.pone.0177360)

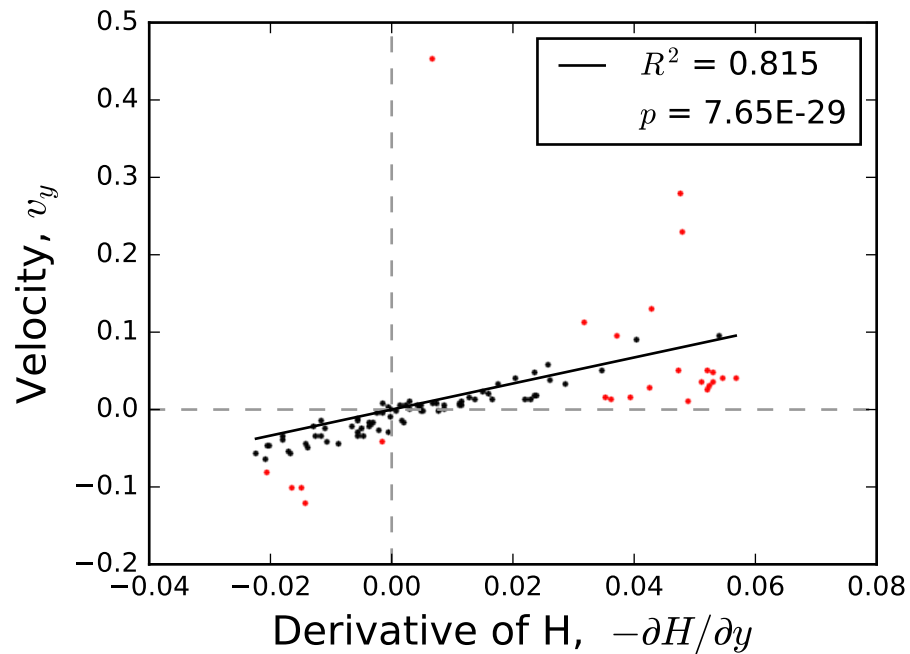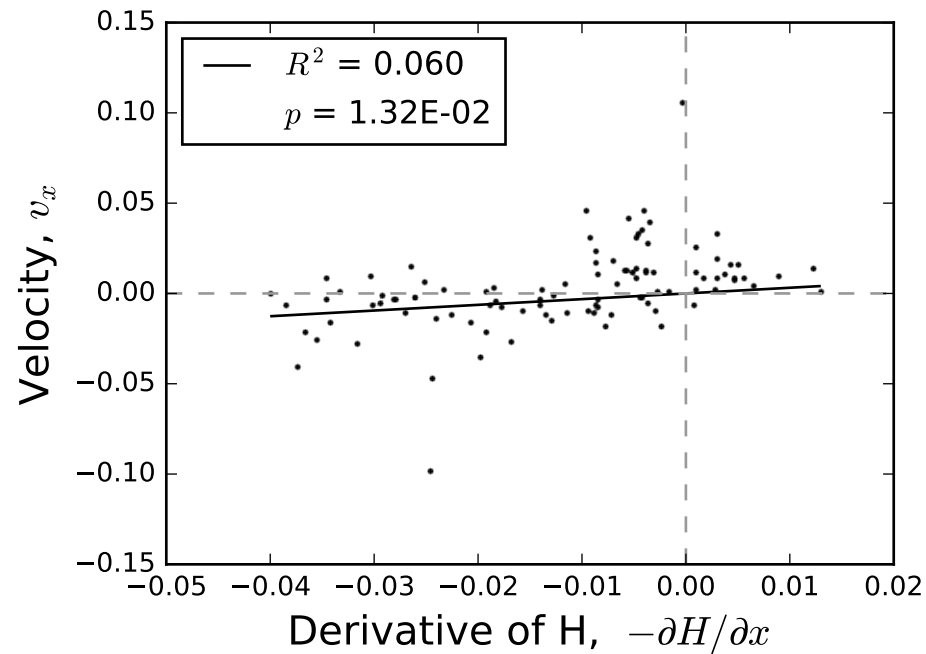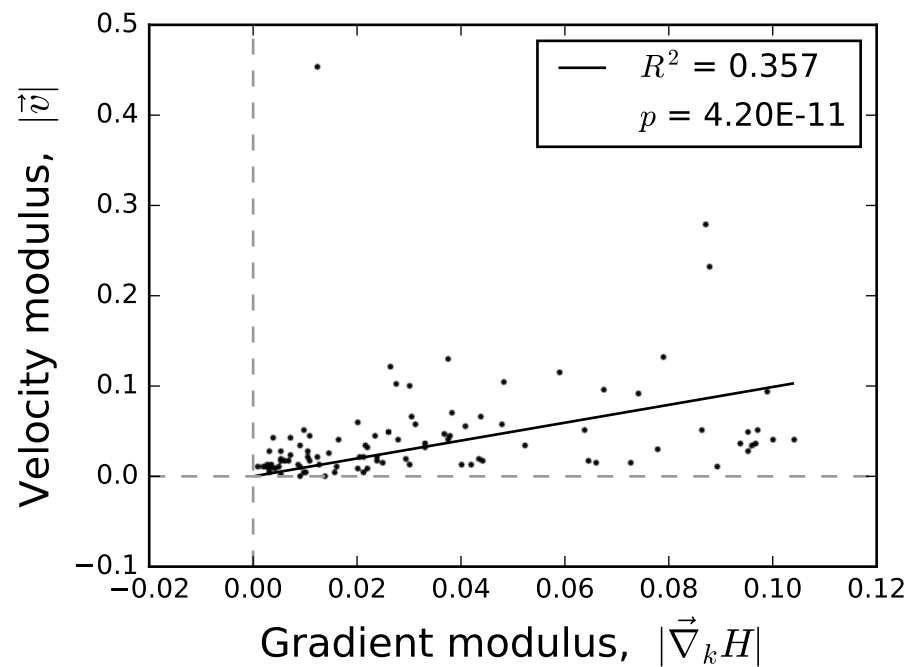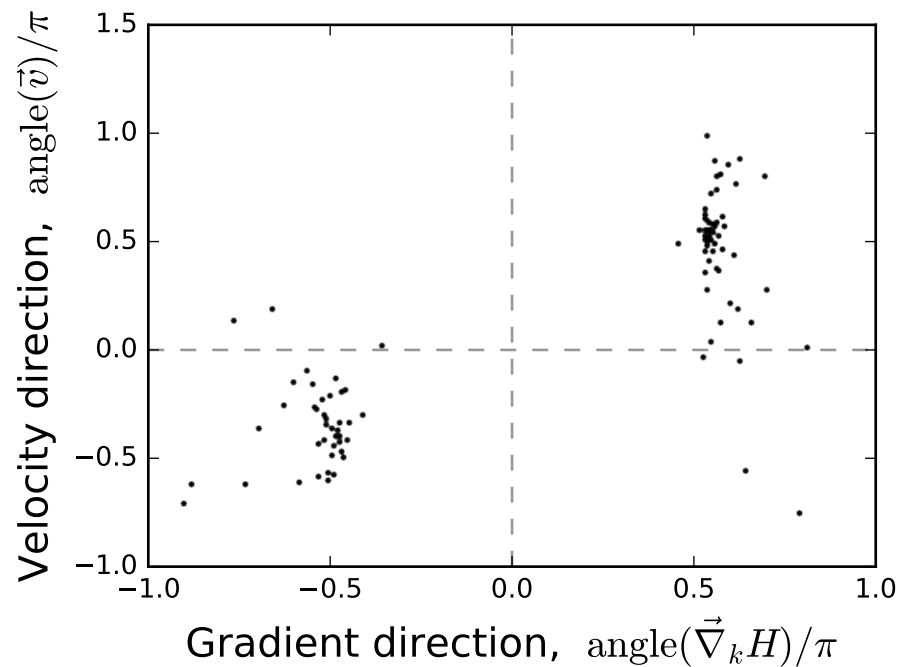

Supplement: S1 Fig — Calculations done on the Feenstra dataset. Top: comparison between the horizontal and vertical components of the field. In the left panel, showing the regression for velocities along the logPRODY axis, the points removed by the RANSAC regression are shown in red. The Bottom: Comparison between orientations and moduli of the field. (PDF) [file pone.0177360.s001.pdf]

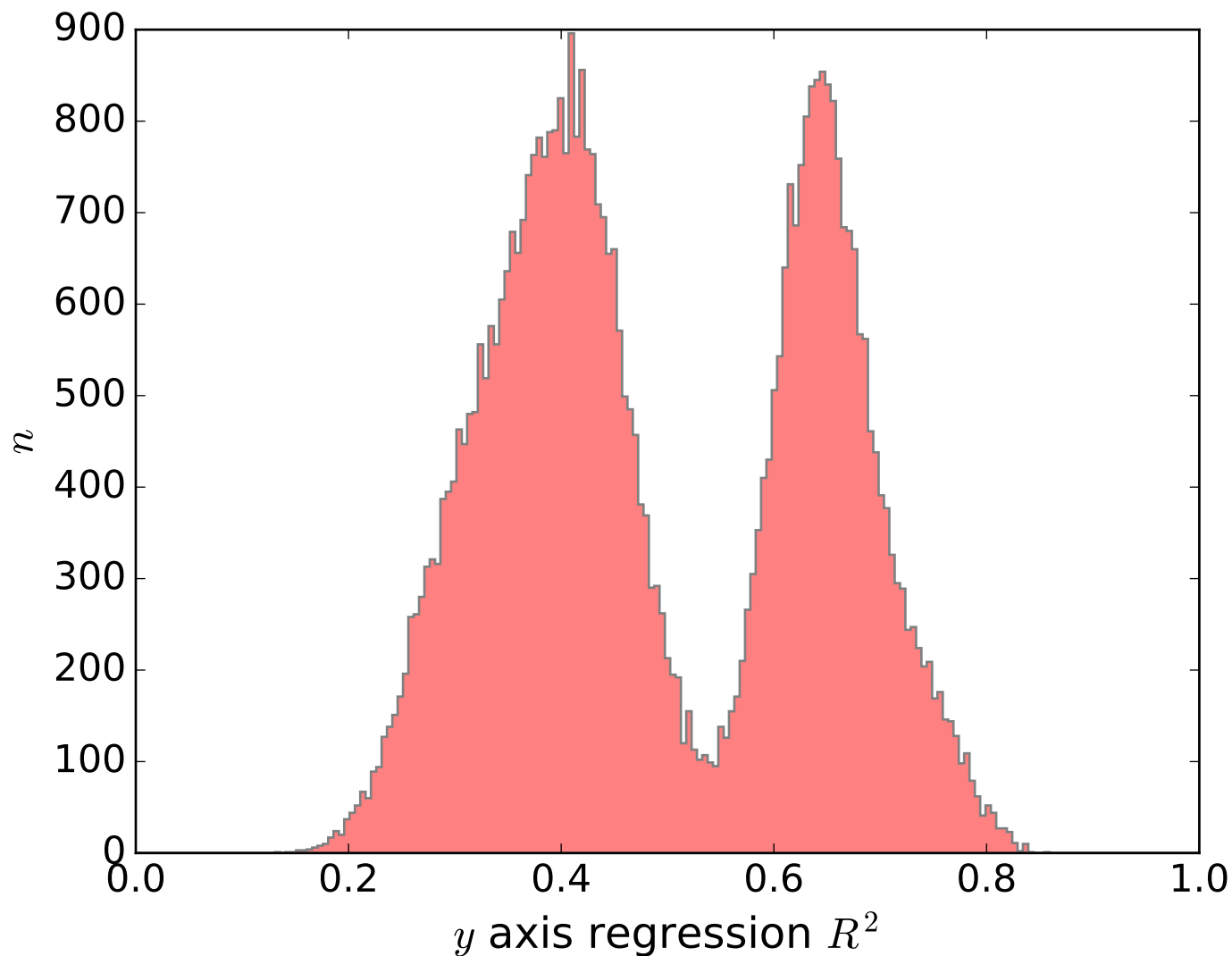

Supplement: S2 Fig — Each iteration of the bootstrap randomly removed 10% of the samples, for 50k iterations. Two peaks are clearly visible: one at about R2 = 4, and one at about .7. The bimodal distribution confirms our hypothesis that the points follow a very clear linear trend, with a few very big outliers. The peak at .7 is caused by the bootstrap randomly removing the outliers from the regression. The peak at .4 comes from the bootstrap removing points following the linear trend, and leaving the outliers in. This clear bimodal distribution allows us to use RANSAC, which is an algorithm apt to take out a few big outliers from a regression. (PDF) [file pone.0177360.s002.pdf]

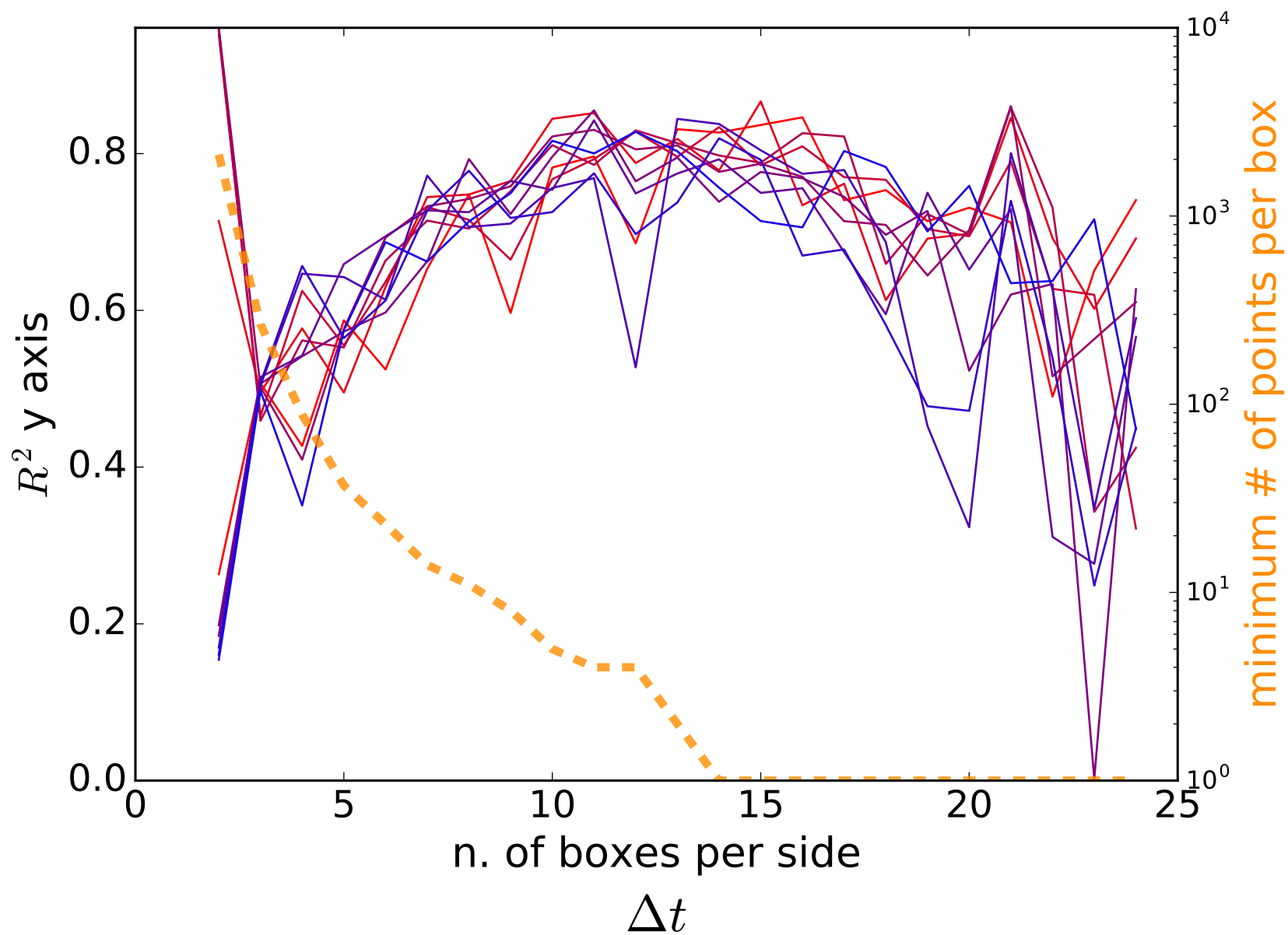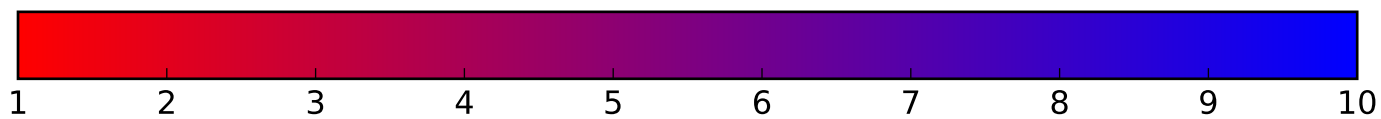

Supplement: S3 Fig — On the horizontal axis there is the resolution of the grid, with the number of boxes per side. In blue to red color, we show the time interval used to calculate the products’ displacements, which are then averaged into v→. The yellow line indicates the minimum number of points per box at a given resolution. The accuracy of our model’s prediction peaks at a resolution between 10 and 15. Less resolution is probably too little detail to capture the features of the system. Accuracy starts to drop, especially for longer time displacements, as soon as the number of points per box is 1; this is probably caused by an increase in noise in the less populated areas of the plane. All the regressions are linear, calculated with RANSAC, and with the zero-order coefficient set to zero by hypothesis. (PDF) [file pone.0177360.s003.pdf]

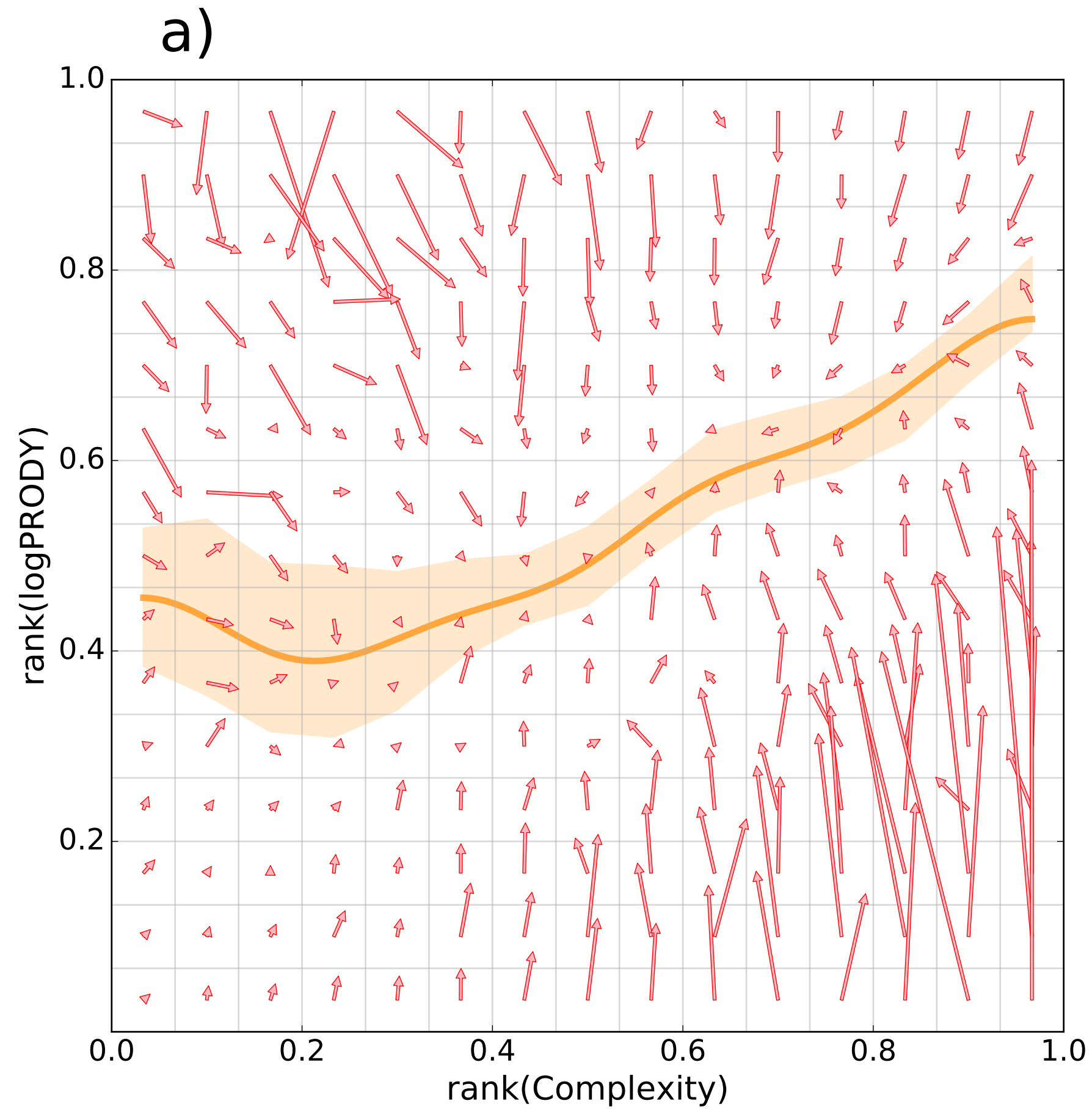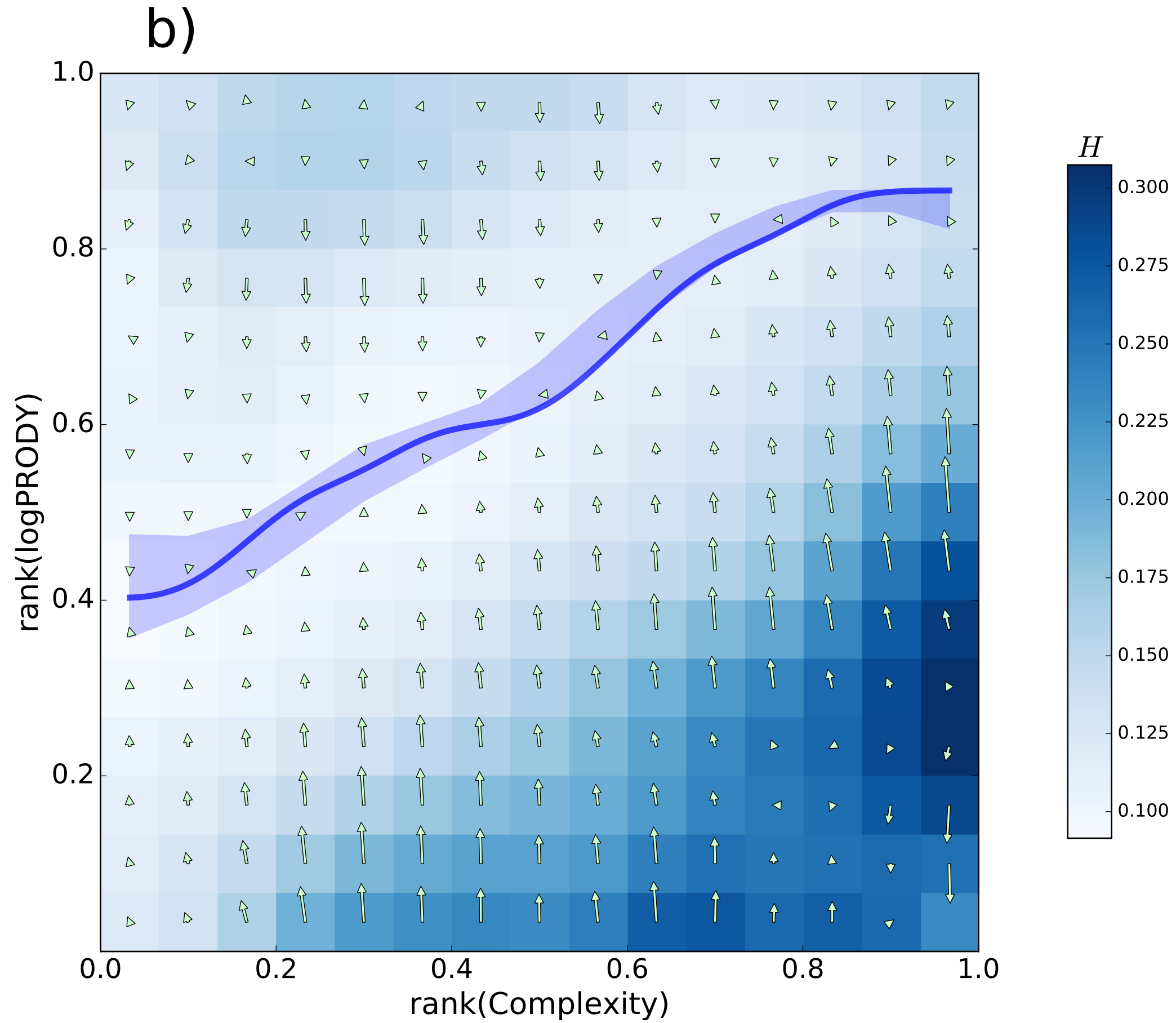

Supplement: S4 Fig — The results are extremely similar to those shown in the paper for the Feenstra dataset, with great consistency across datasets. Panel a). In red, the average velocity field of the products on the RCLP plane, v→. The asymptotic zone is marked by the orange line, together with the bootstrap result for the 5% confidence interval. Panel b). Average Herfindahl per box, the H field (in blue), with the derivatives (green arrows). The line is the kernel regression of the minima of the Herfindahl field per column, with 5% confidence interval on the value superimposed. All the arrows in both panels are to 1:1 scale with each other and with the plot’s axes. (PDF) [file pone.0177360.s004.pdf]

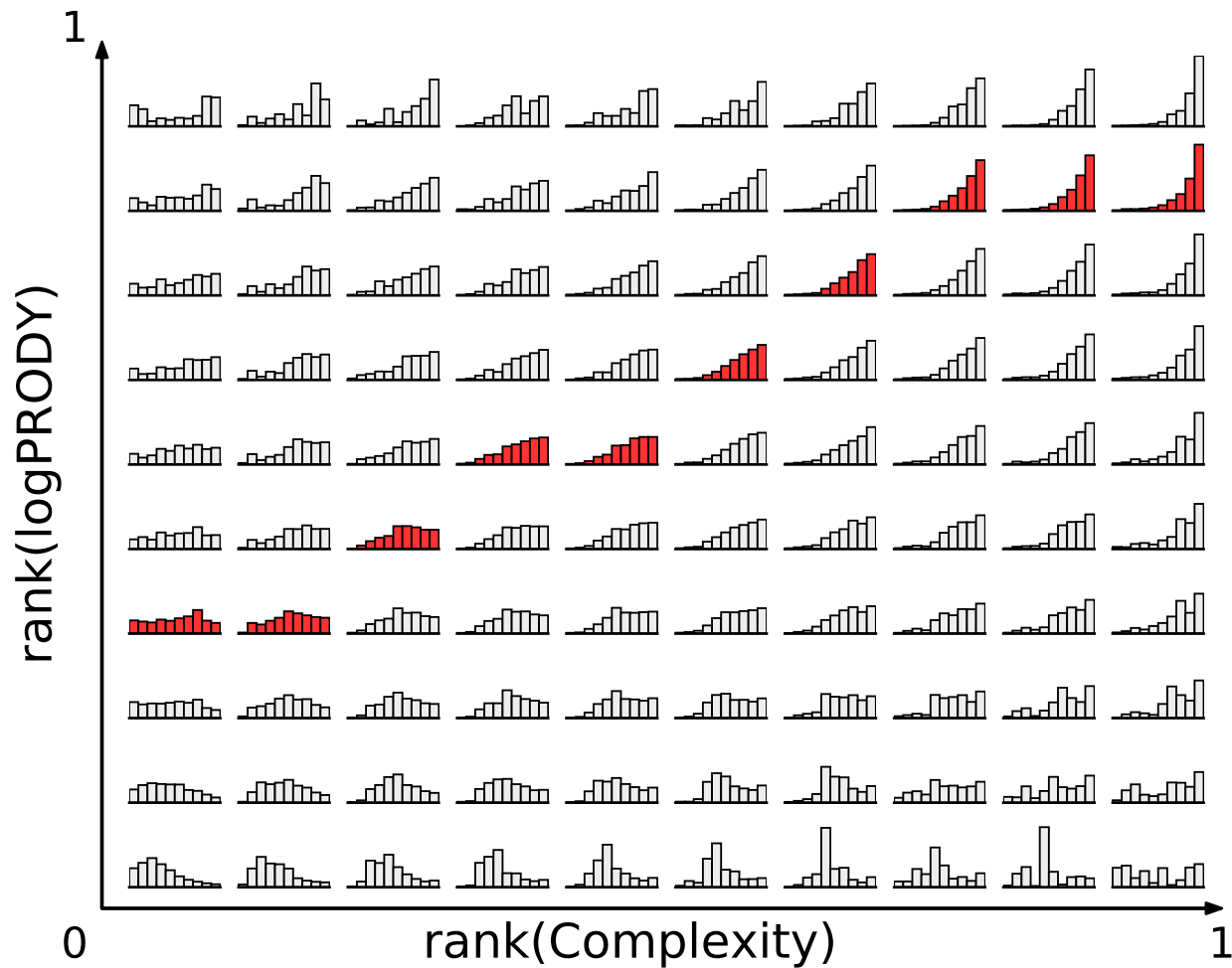

Supplement: S5 Fig — For each box b we plot a histogram showing the average R˜ values of countries exporting the products contained in b. Each bar of the histograms shows the average RCA of countries with a fitness value between two consecutive deciles of the fitness distribution. We remind that R˜ represents the share of a product in a country’s total exports. Here we see the same patterns found in the Feenstra dataset: the distributions on the minima of H going from flat for the lowest Complexity level to markedly peaked on high fitness for the highest Complexity. Again, the results show consistency. (PDF) [file pone.0177360.s005.pdf]

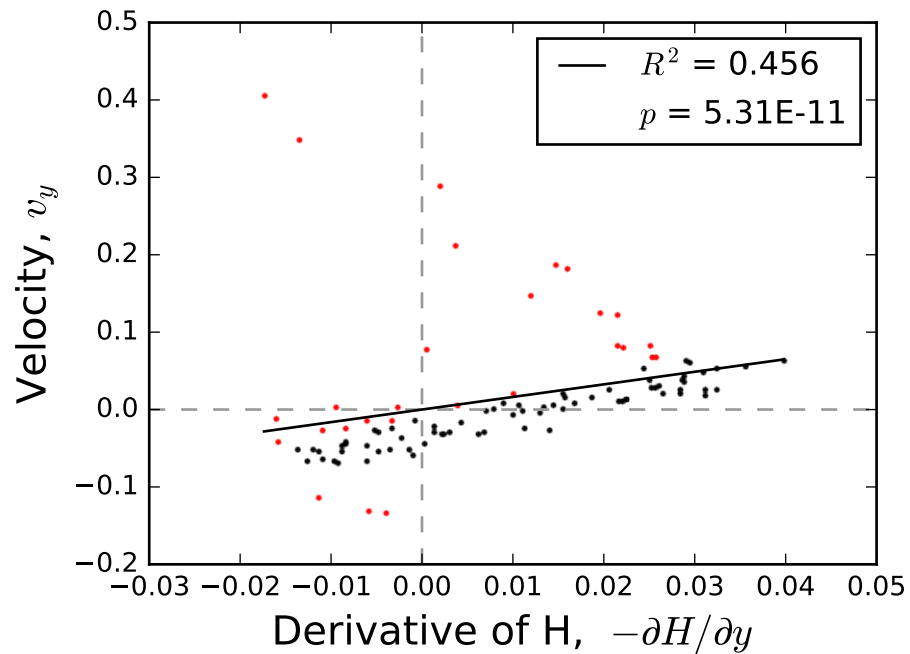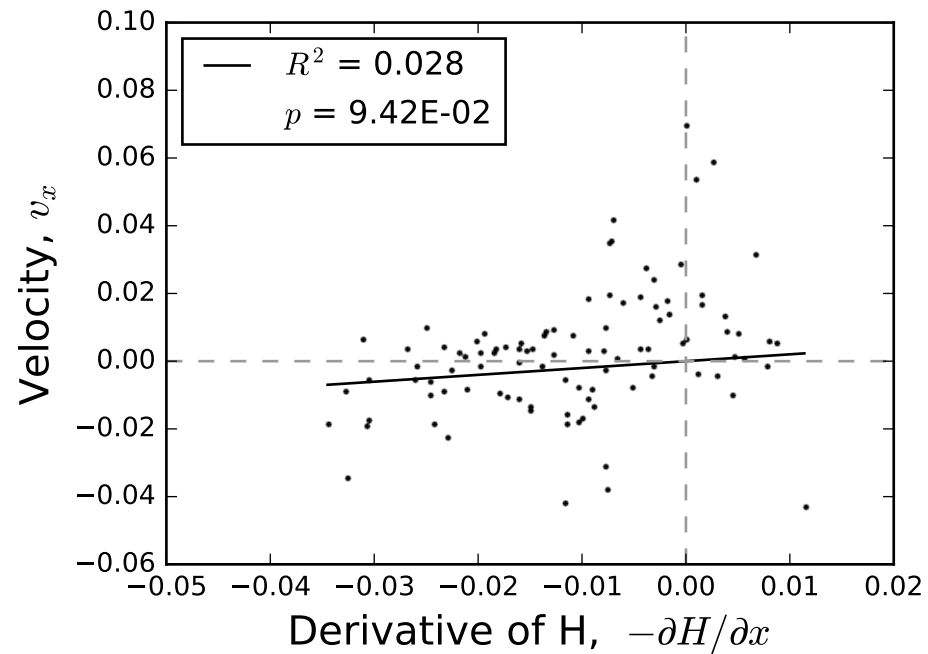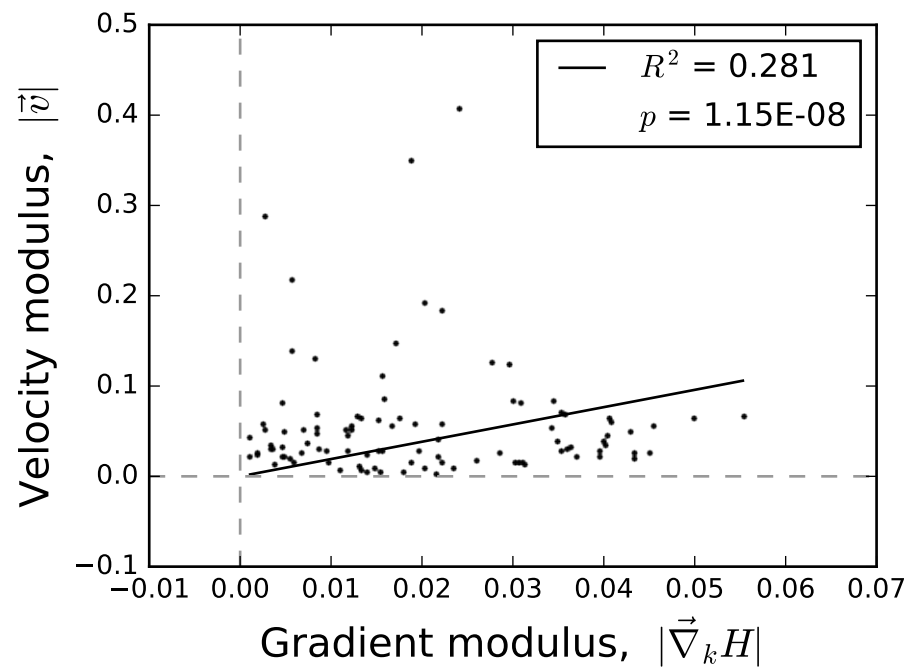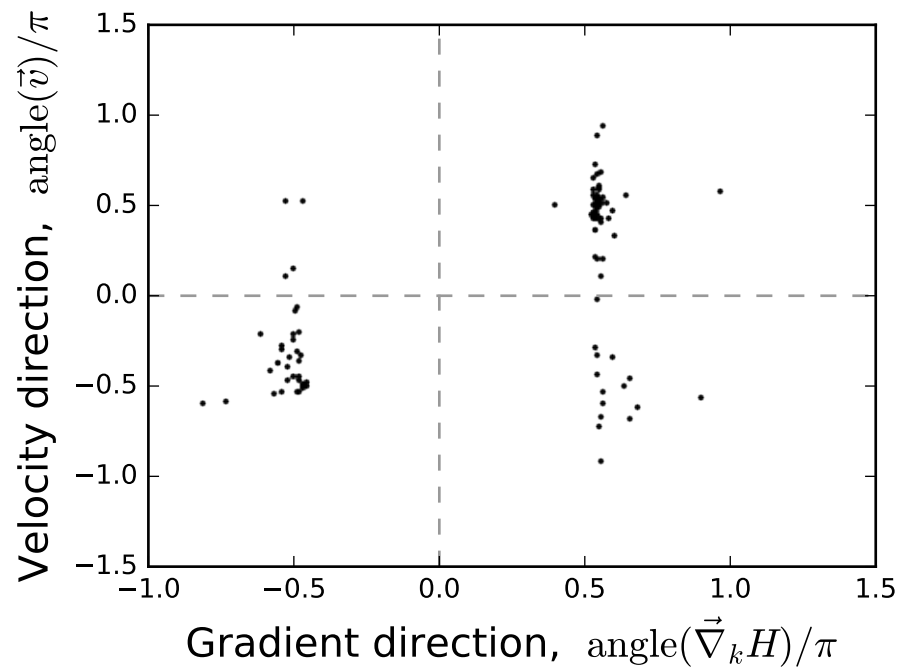

Supplement: S6 Fig — Calculations done on the BACI dataset. Top: comparison between the horizontal and vertical components of the field, the latter regressed with RANSAC. Bottom: Comparison between orientations and moduli of the field. (PDF) [file pone.0177360.s006.pdf]

a)

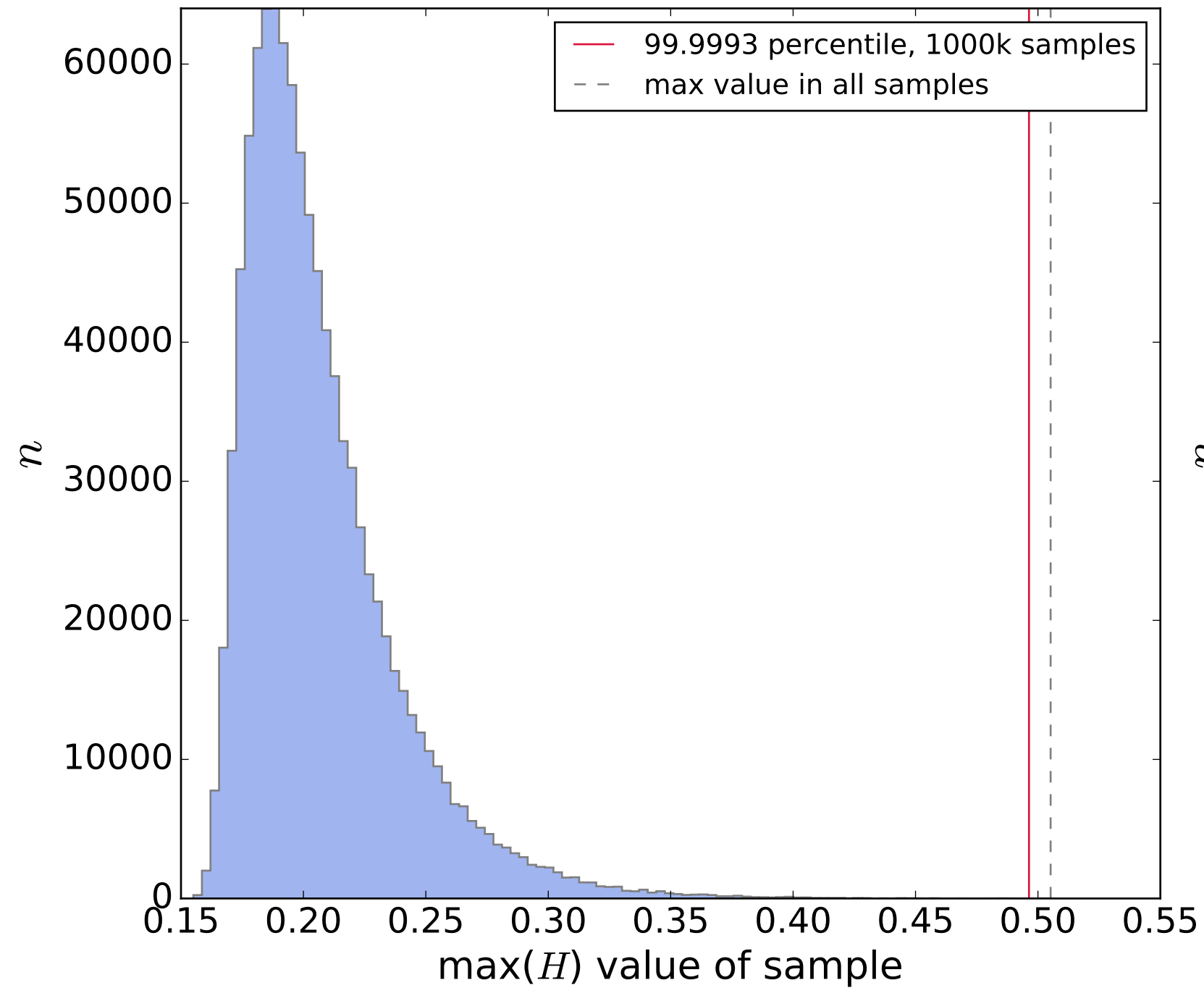

b)

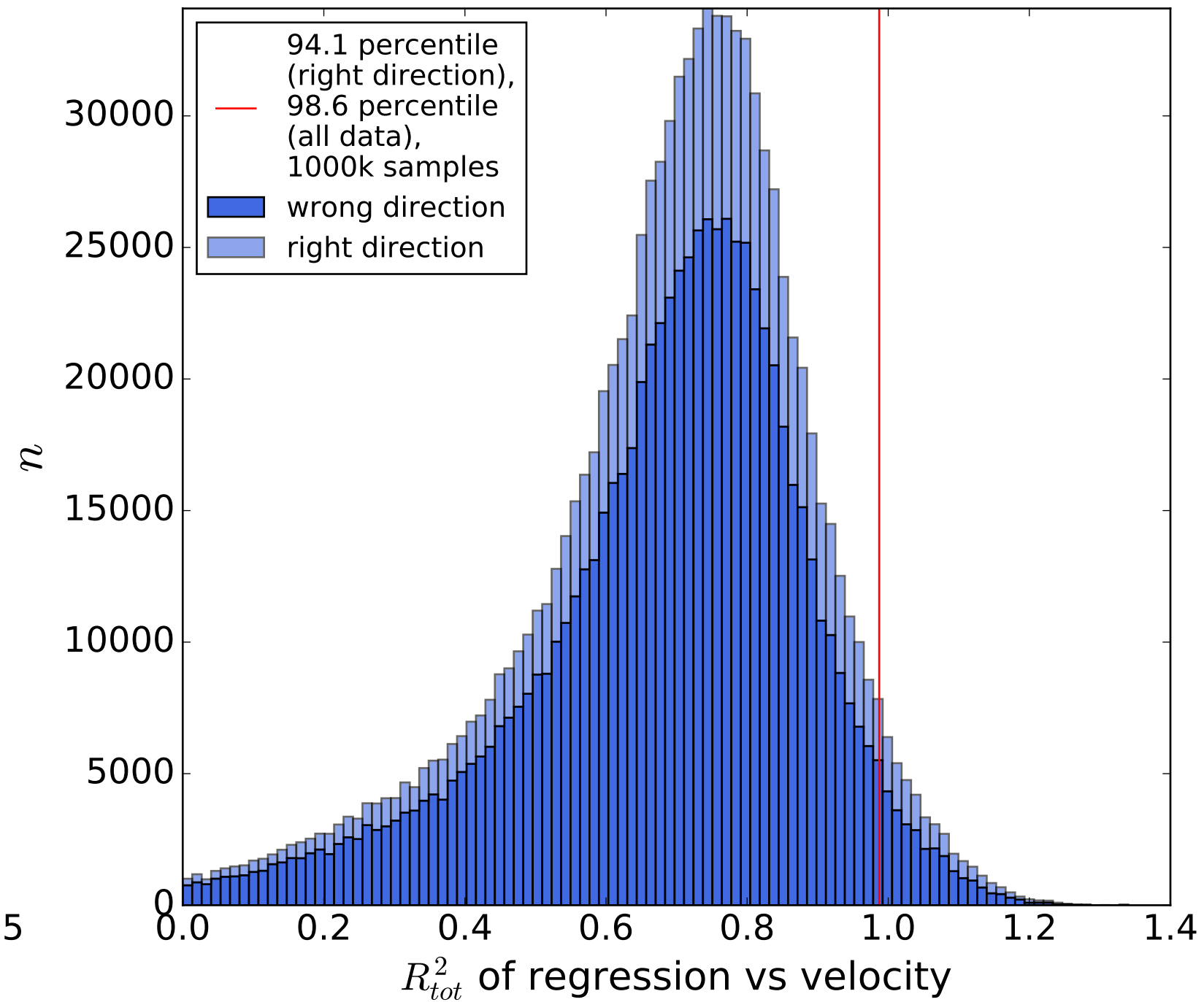

Supplement: S8 Fig — Panel a) Maximum value of the H field. Given that the less points one has per box, the higher the standard deviation for H in that box is, one expects to find higher values of H where the density of points is lowest on the RCLP plane. We check whether the null model can produce a H˜ field with the same highest value found in H, which we will call max H. Running the model 1M times produced few samples of comparable highest value max H˜. In the histogram we show the frequency of max H˜; the vertical line shows max H for the Feenstra dataset. A similar result (not shown) is found in the BACI dataset. Panel b) Check of the potential-like equation’s validity versus the null model. We ran the null model 1M times, and for each H˜ field obtained we calculated the correlation between H˜’s and v→’s vertical and horizontal components with a linear regression. The sum of the two Pearson’s R2 coefficients obtained from this process, Rtot2 (which is a number between 0 and 2), is used here as a measure of significance for the H˜. In this histogram we show the frequency of the Rtot2 values; the vertical line represents Rtot2 obtained from the H field. The null model produces gradients that correlate both positively or negatively with each component of v→: in some cases, one gets a high Rtot2, but the field being reproduced is the inverse of v→. In light blue, we show the fraction of the H˜’s that correctly reproduce the direction of v→ on both components, while dark blue represents the fraction that reproduces at least one of the components of -v→. The result is that, among all generated H˜ fields (both with right and wrong direction), those with a significance level higher than that of H are 1.5%. (PDF) [file pone.0177360.s008.pdf]

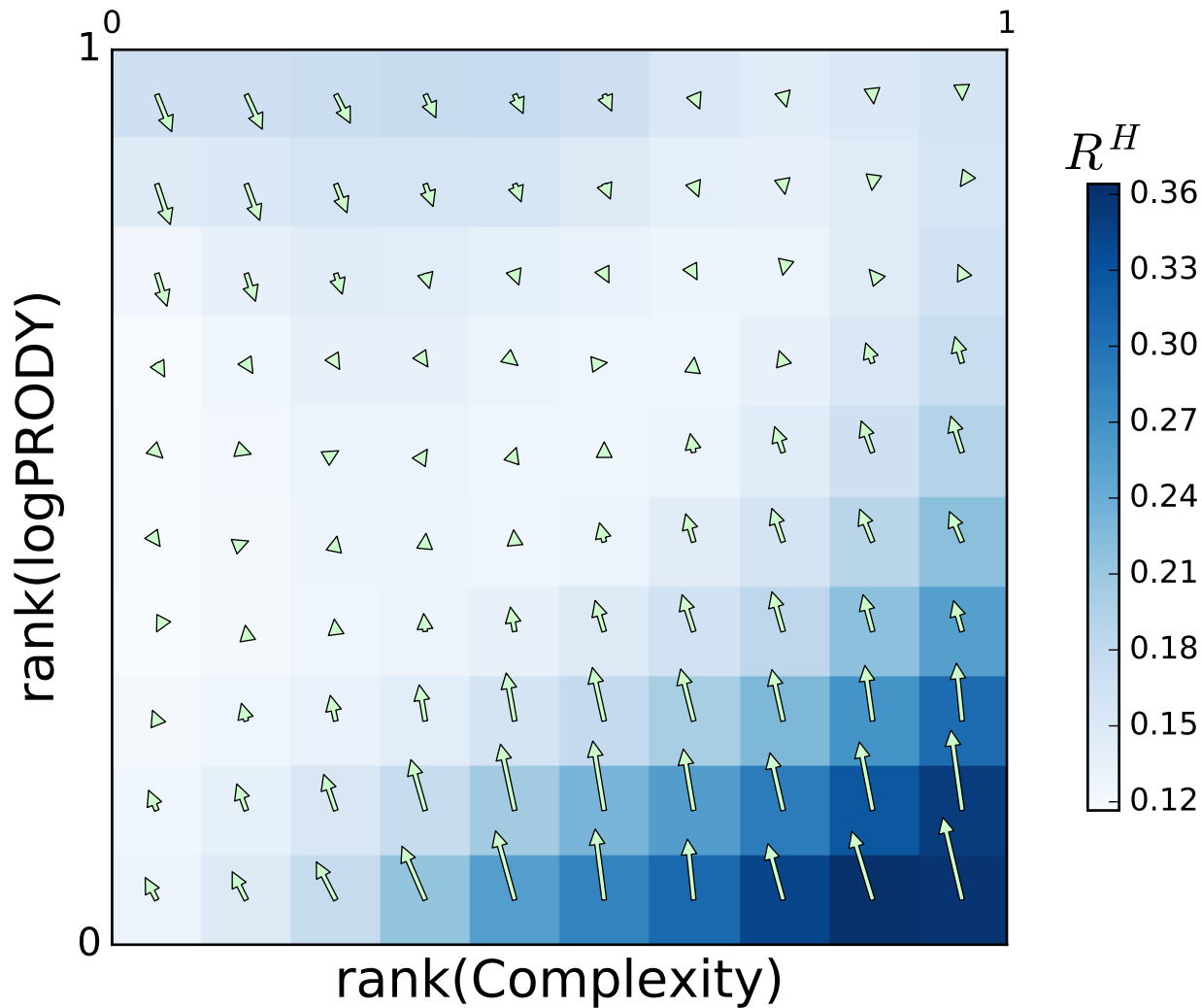

Supplement: S9 Fig — (PDF) [file pone.0177360.s009.pdf]

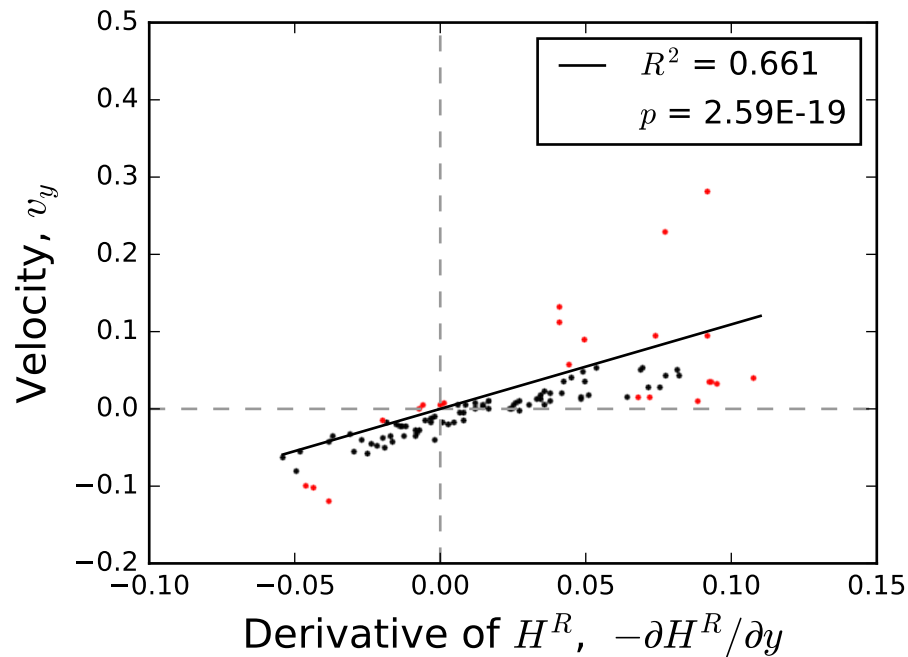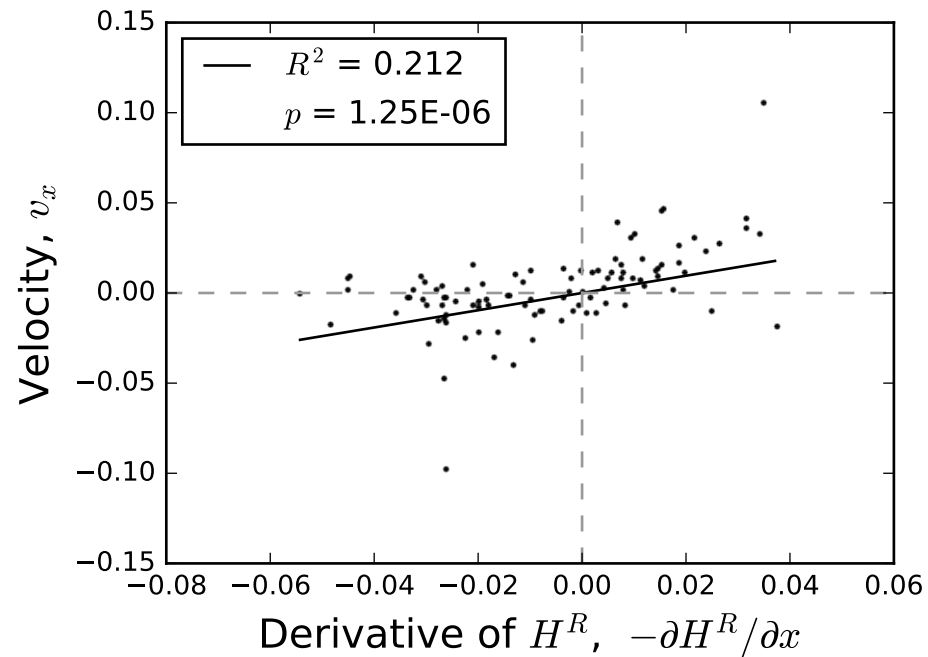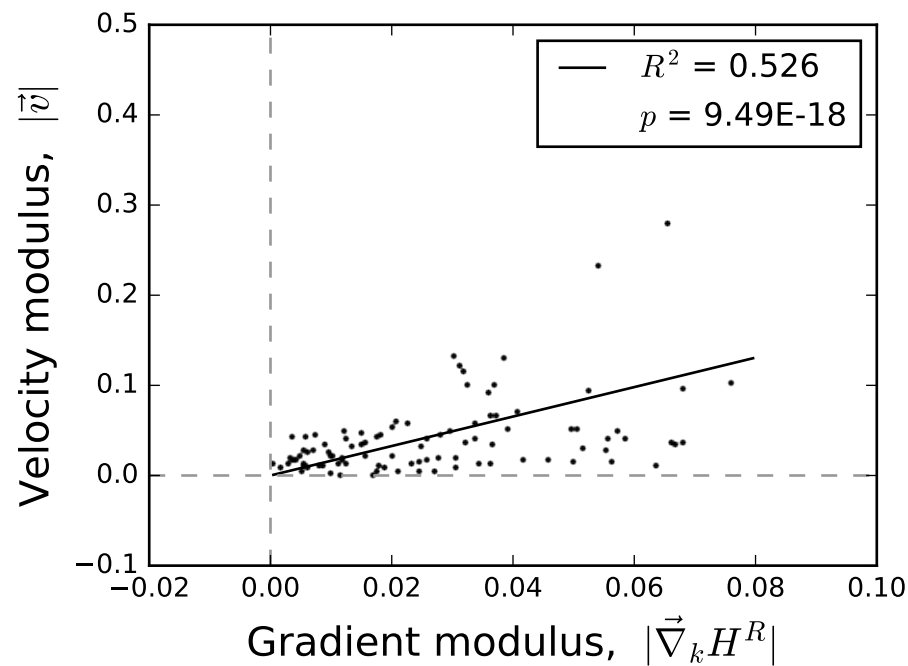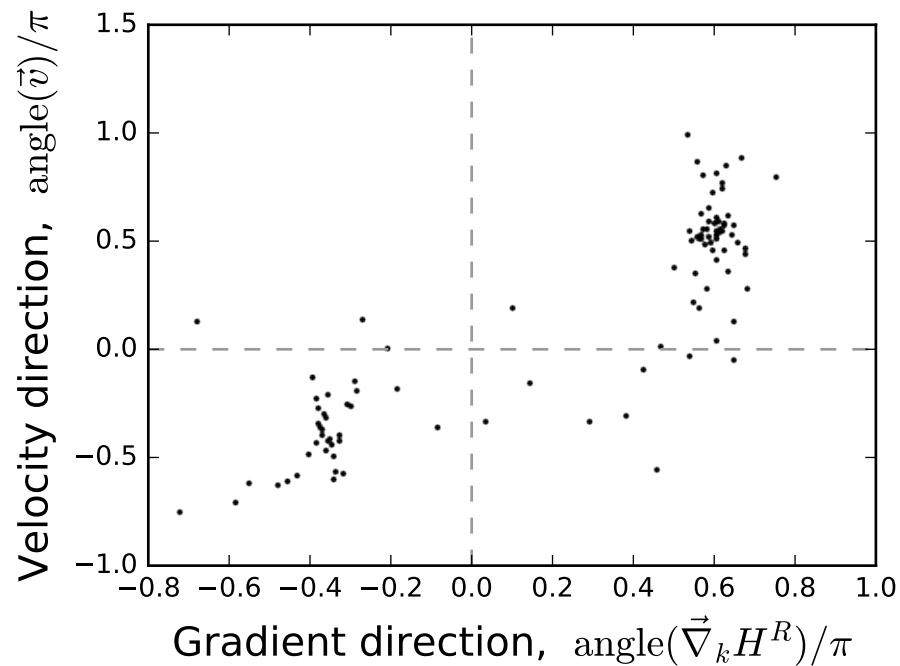

Supplement: S10 Fig — Calculations done on the Feenstra dataset. Top: comparison between the horizontal and vertical components of the field Bottom: Comparison between orientations and moduli of the field. (PDF) [file pone.0177360.s010.pdf]

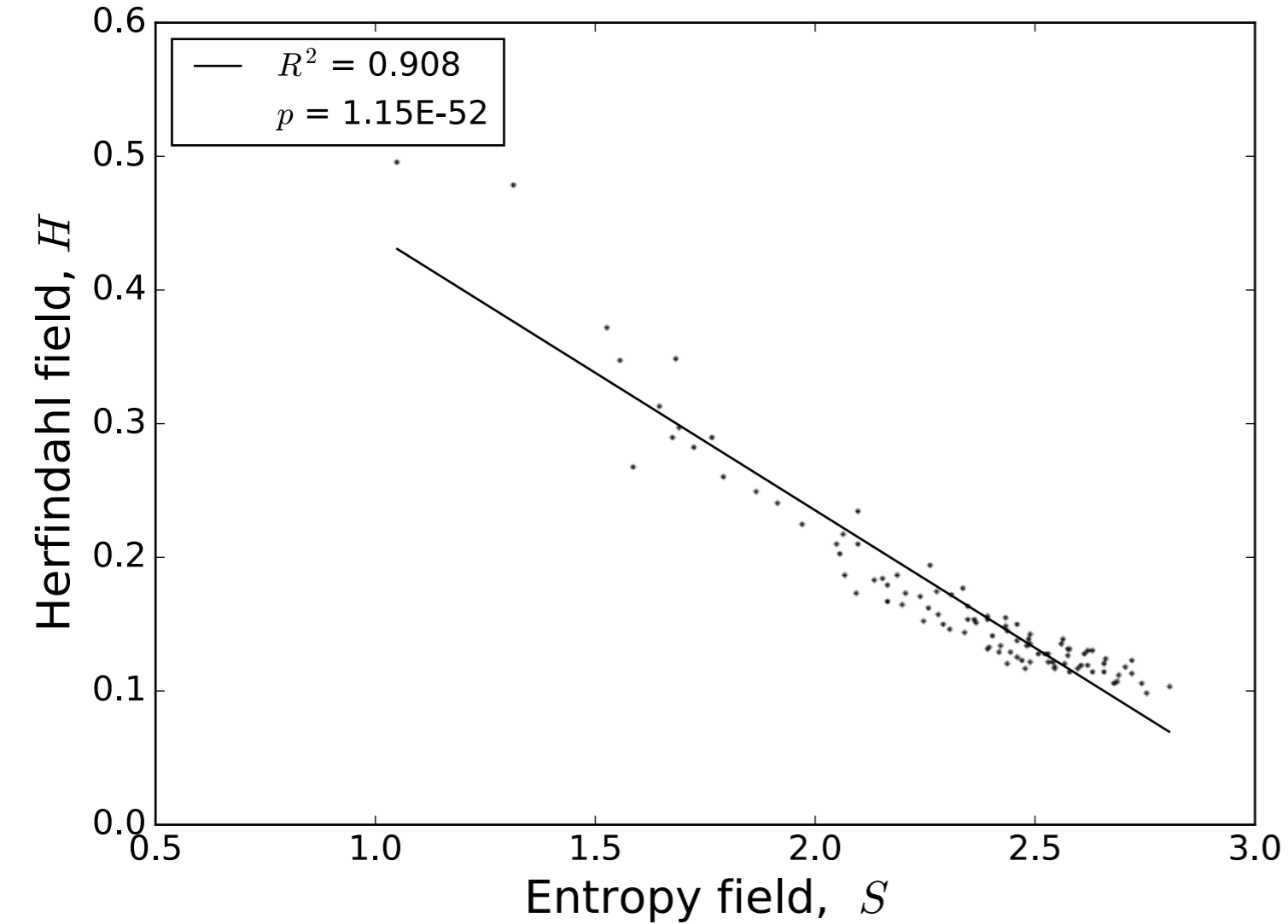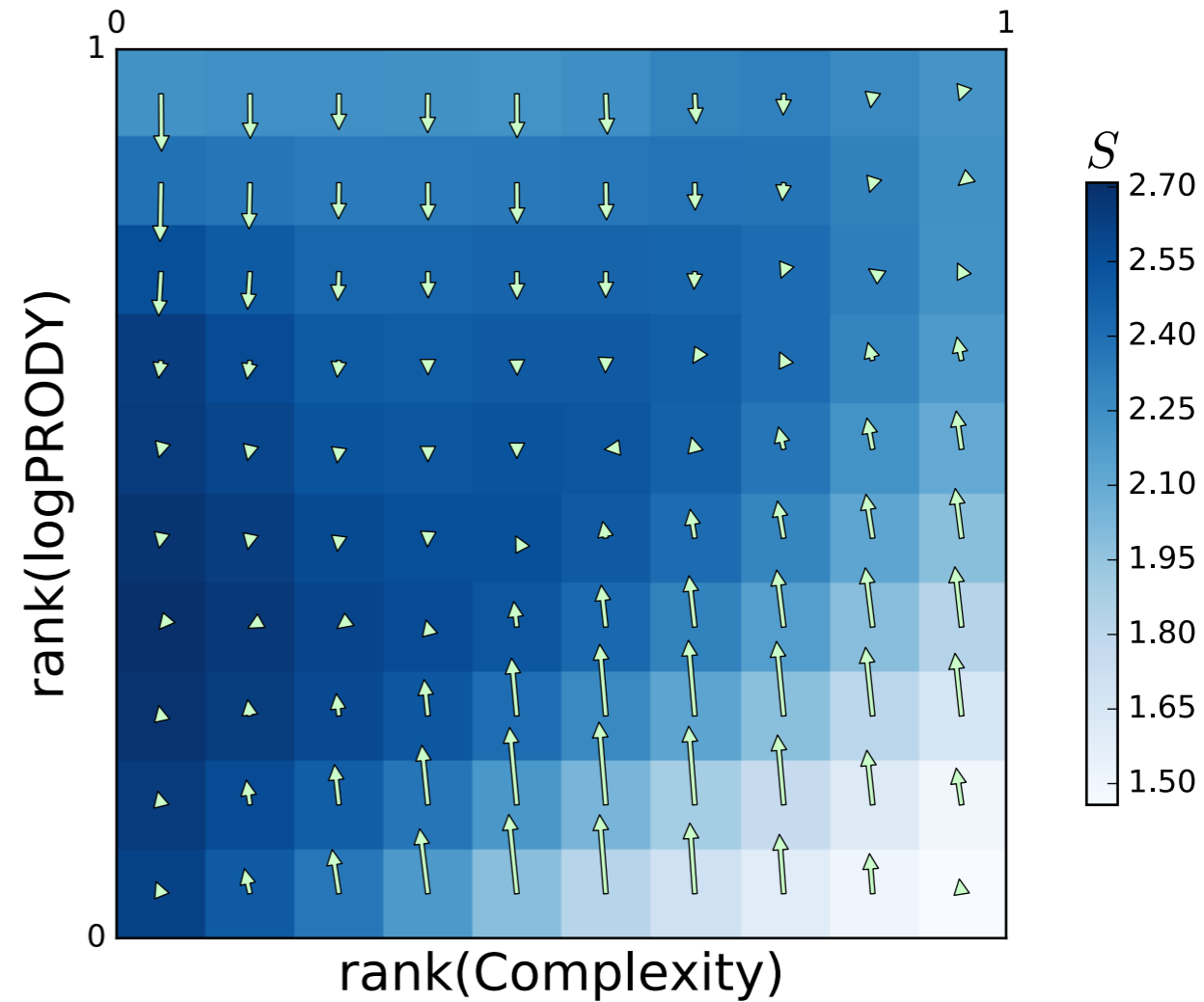

Supplement: S11 Fig — Left: Relation between values of the S and H field in the BACI dataset. Left, in black, a linear fit, which is good enough for the fields, that aggregate many individual points. The actual relationship between entropy and Herfindahl index of a distribution is not linear, as we show in the next section. Right: The field obtained by using S. (PDF) [file pone.0177360.s011.pdf]

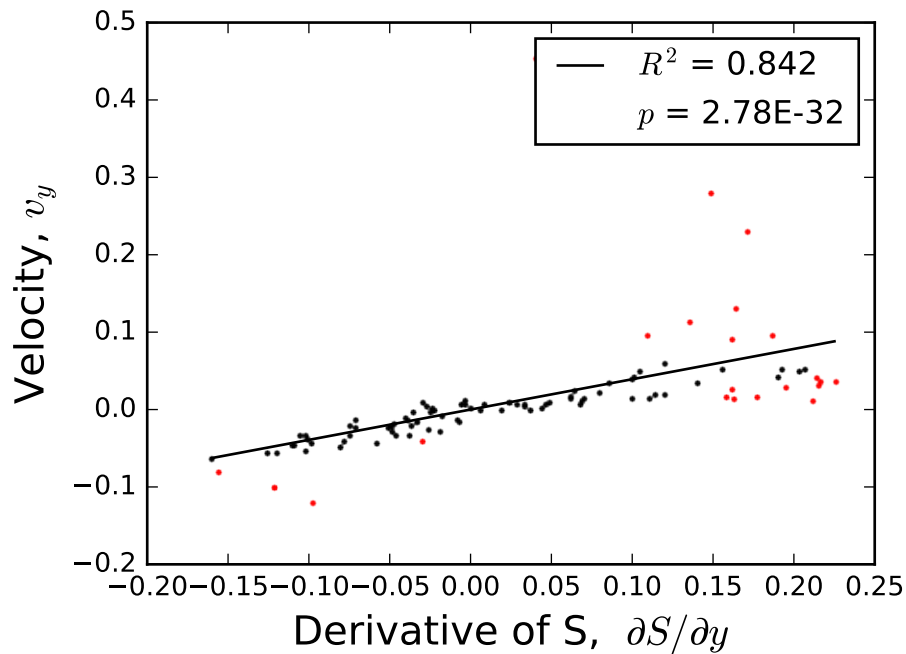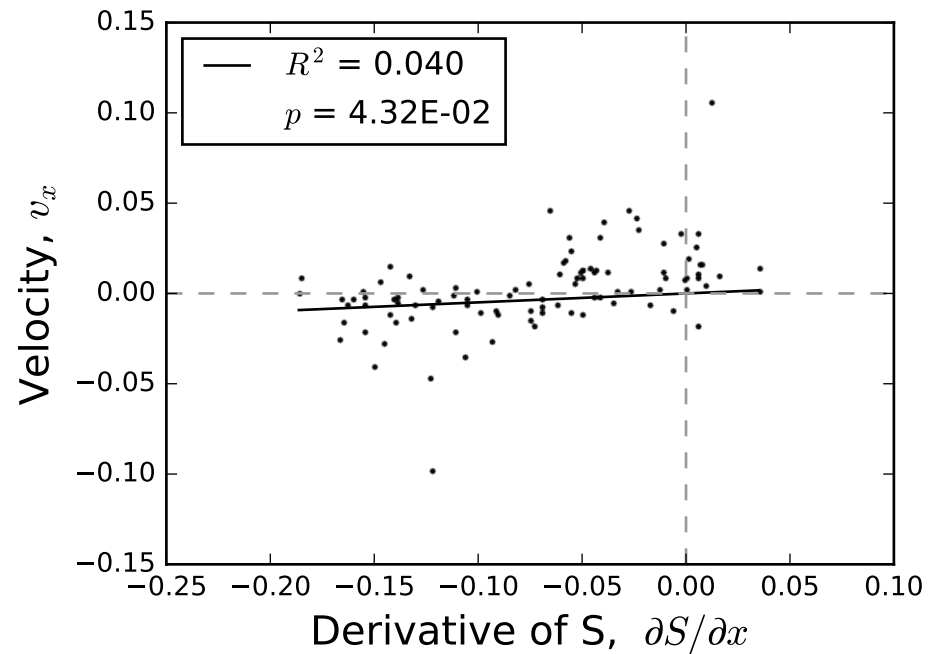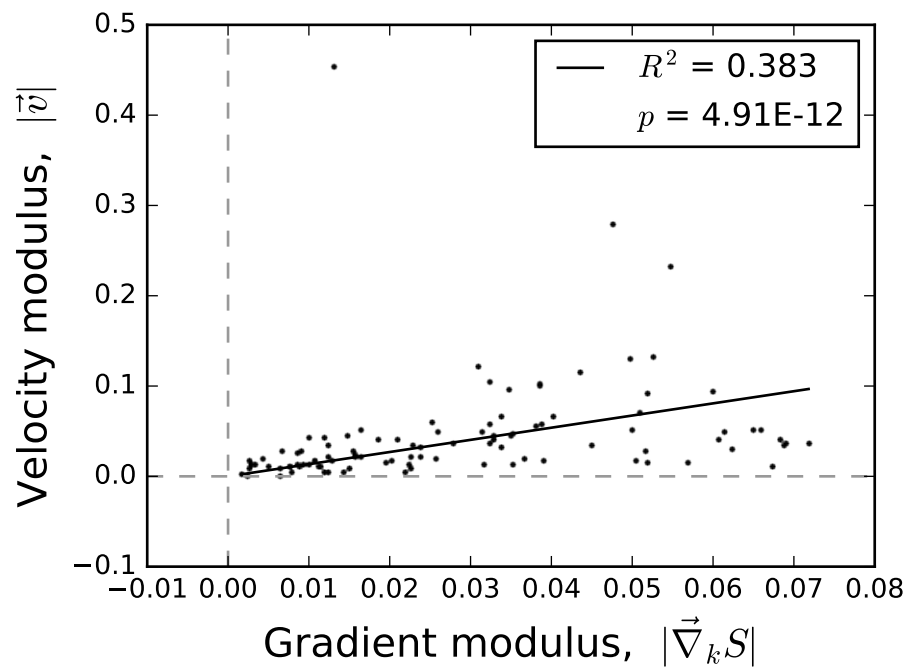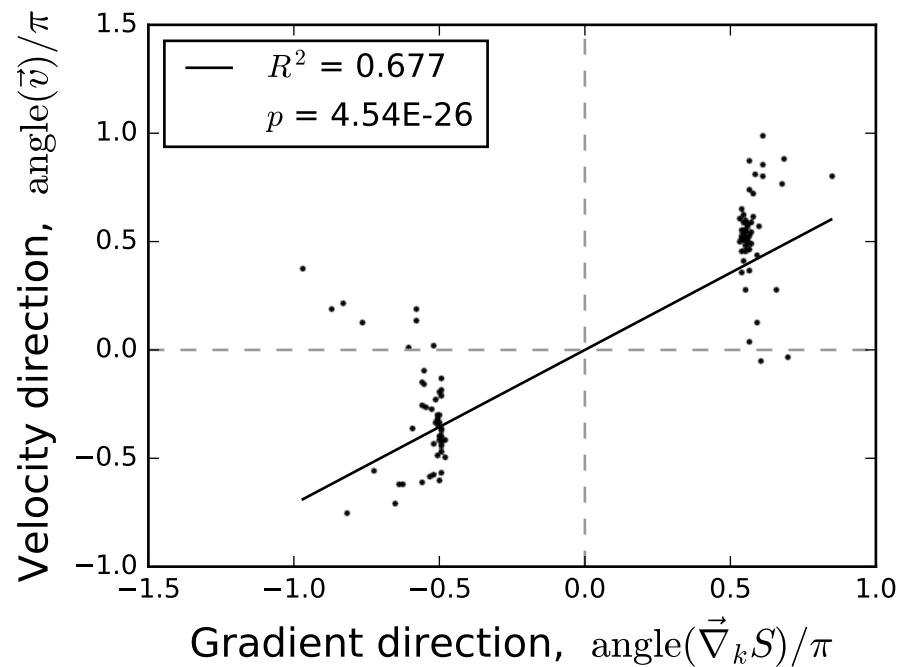

Supplement: S12 Fig — Calculations done on the Feenstra dataset. Top: comparison between the horizontal and vertical components of the field Bottom: Comparison between orientations and moduli of the field. (PDF) [file pone.0177360.s012.pdf]

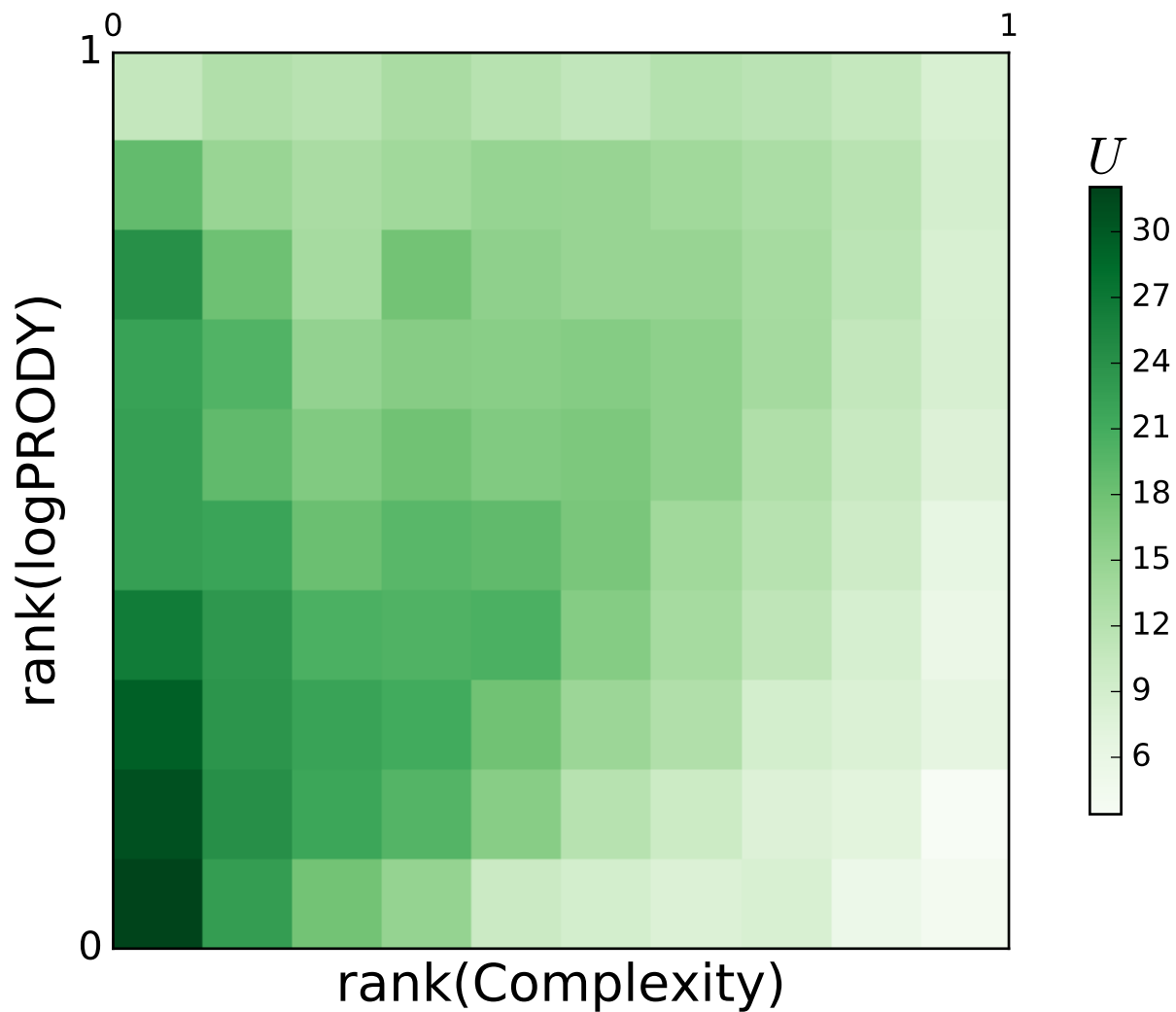

Supplement: S13 Fig — It is different from the field obtained by regressing H, because the average Ubiquity of products decreases with increasing Complexity; therefore the left part of the RCLP plane has higher values than the right. The plot shows the regression for the Feenstra dataset. (PDF) [file pone.0177360.s013.pdf]

average inwards velocity field

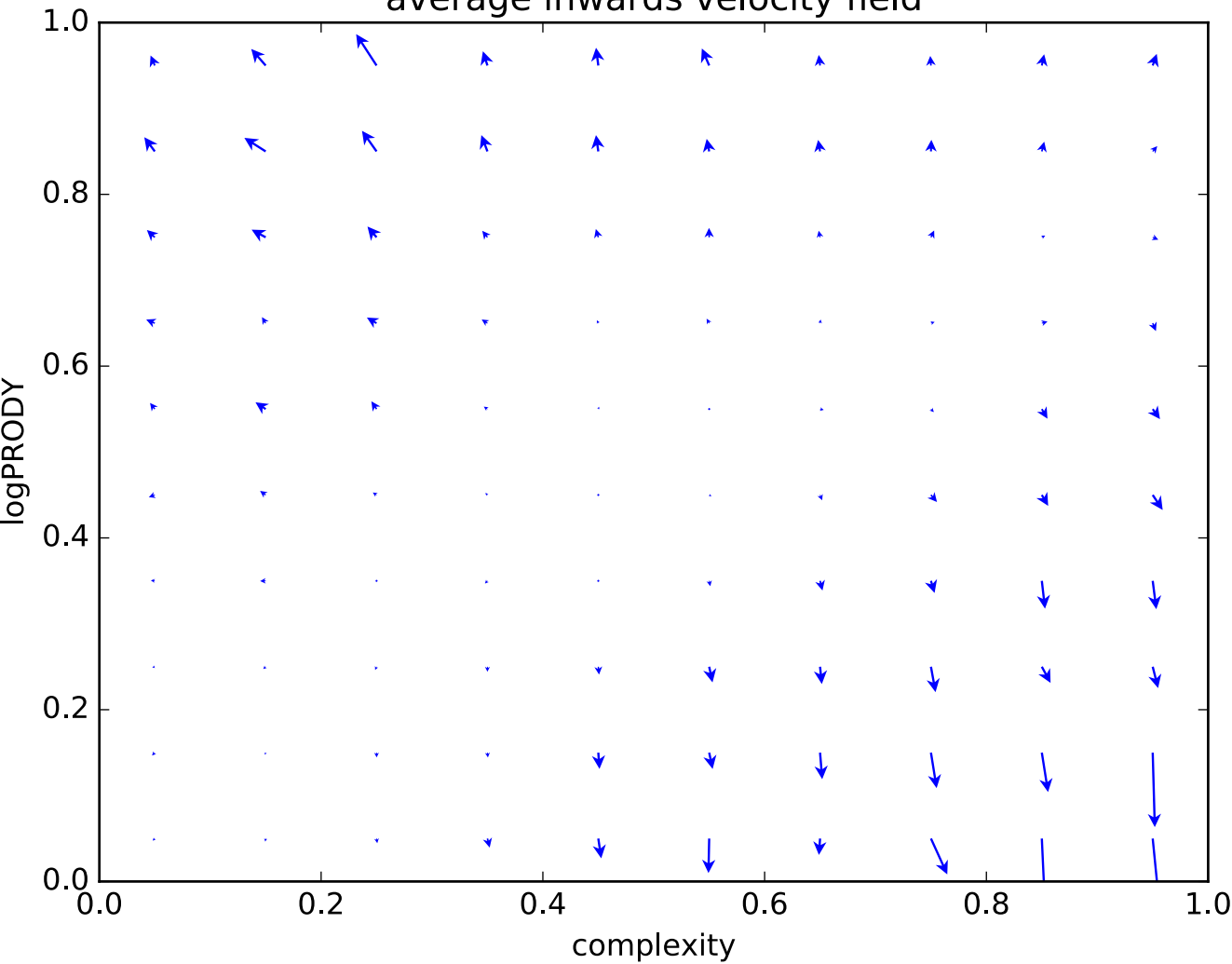

Supplement: S14 Fig — To calculate this field, considered all products that entered a given box, and averaged their displacements. The vectors are not to scale with those shown in the rest of the paper. To allow comparison, we present a depiction of both w→ and v→ fields in the next figure. The figure refers to the BACI dataset. (PDF) [file pone.0177360.s014.pdf]

r=outwards, b=inwards speed, to scale

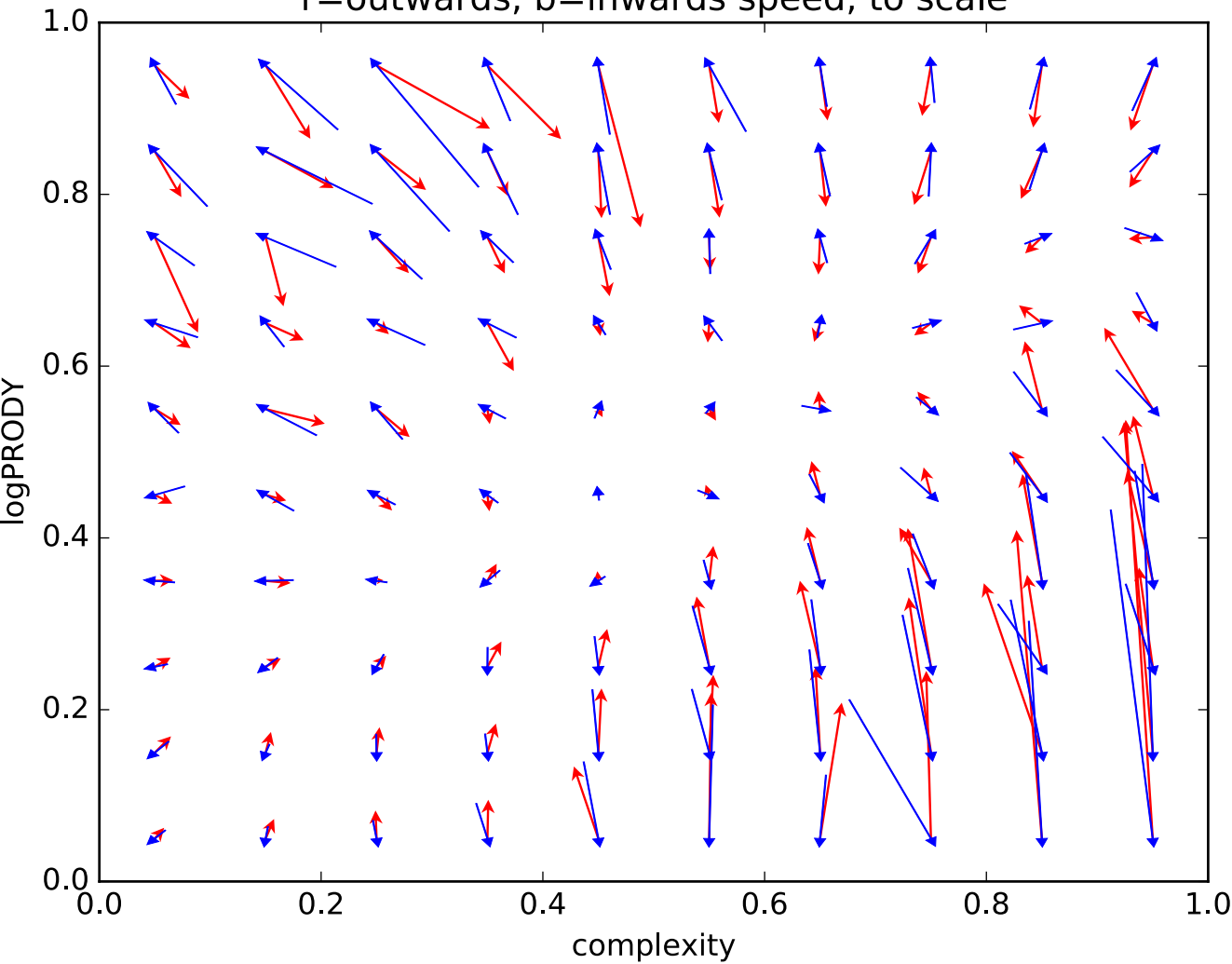

Supplement: S15 Fig — The figure refers to the BACI dataset. (PDF) [file pone.0177360.s015.pdf]

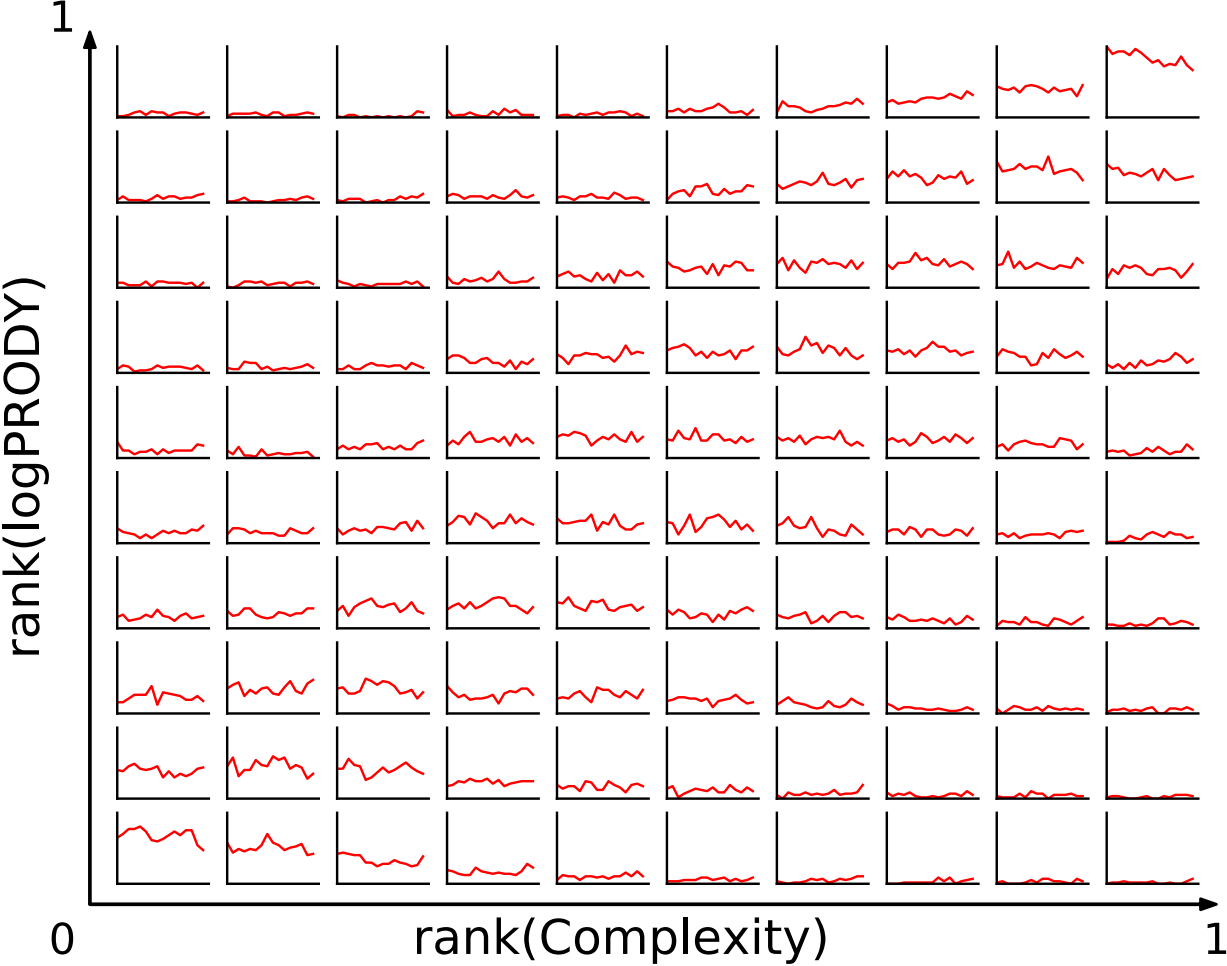

Supplement: S16 Fig — Each of the plots in this figure shows the evolution in the number of products contained in each box. The horizontal axis of the plots represents time in years, and the vertical axis the number of points. The plots are to scale relative to each other. The figure refers to the BACI dataset. (PDF) [file pone.0177360.s016.pdf]

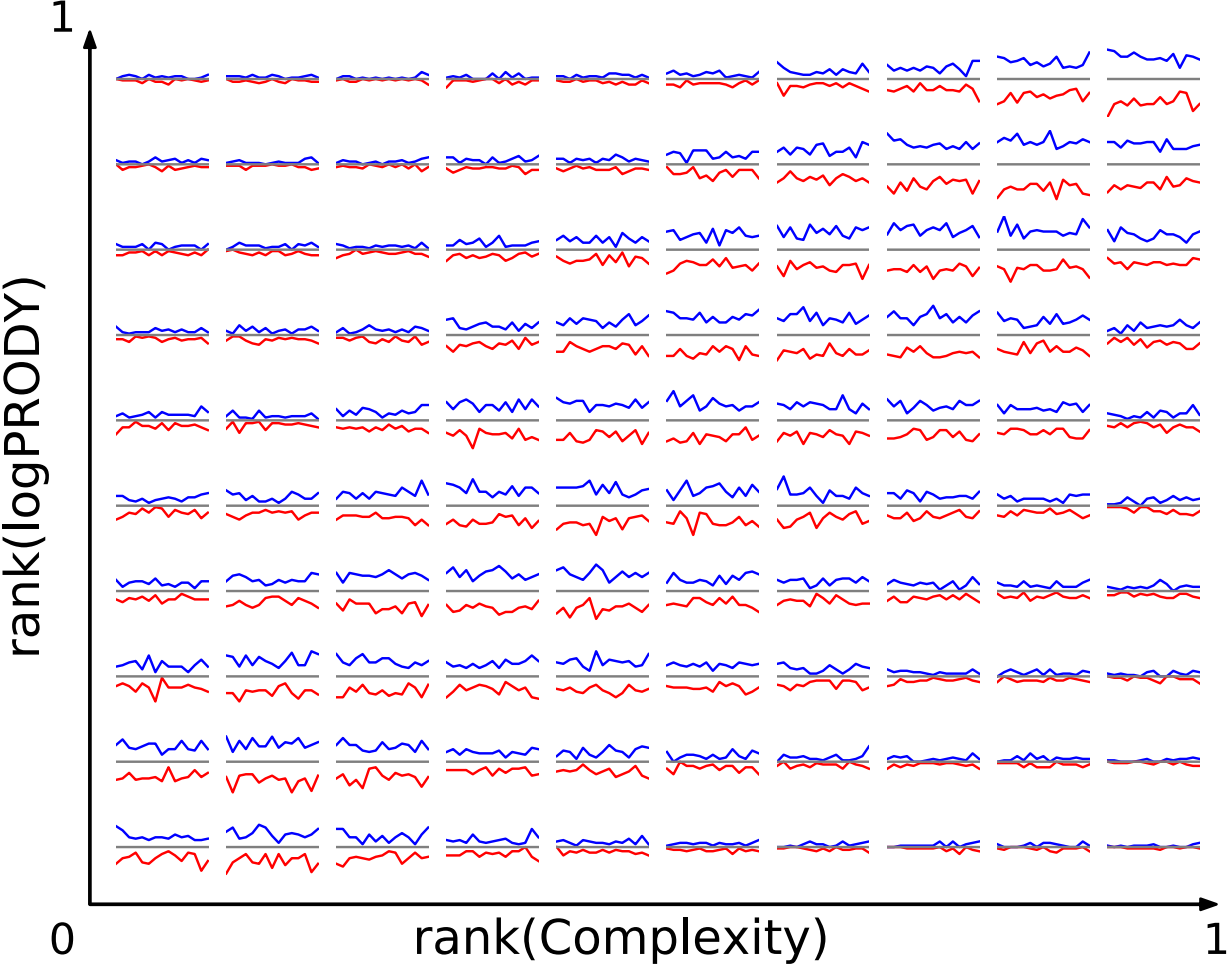

Supplement: S17 Fig — Each of the plots in this figure shows the yearly change in the number of products contained in each box. The horizontal axis of the plots represents time in years, and the vertical axis the difference in number of points (for the outward flux the difference is negative). The plots are to scale relative to each other. The figure refers to the BACI dataset. (PDF) [file pone.0177360.s017.pdf]

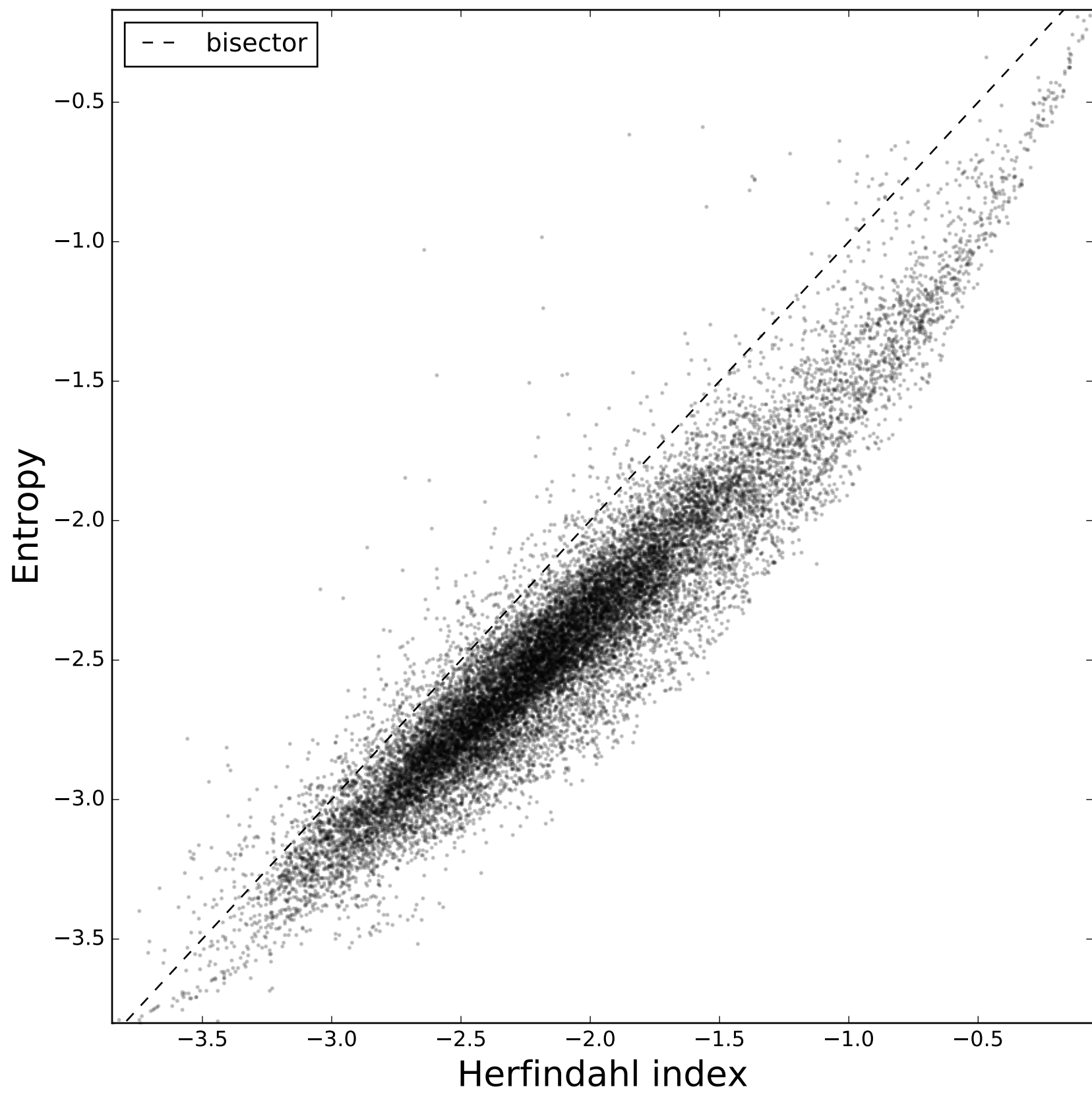

Supplement: S18 Fig — (PDF) [file pone.0177360.s018.pdf]
