# Supplementary material for: Correction: The complex dynamics of products and its asymptotic properties
Source: PLoS One. 2017 Oct 11;12(10):e0186436. doi: 10.1371/journal.pone.0186436 (PMC5636154; doi:10.1371/journal.pone.0186436)
Supplement: S19 File — Here we report and discuss a number of auxiliary results supporting the main findings of the principal paper and assess the robustness of our calculations. (PDF) [file pone.0186436.s001.pdf]

SUPPLEMENTARY INFORMATION - The complex dynamics of products and its asymptotic properties

Orazio Angelini[1],**Matthieu Cristelli**[1],**Andrea Zaccaria**[1],**Luciano Pietronero**[1,2]

[1]ISC-CNR, Institute for Complex Systems, Rome, Italy

[2]Physics Department, Sapienza University of Rome, Rome, Italy

E-mail: `angelini.orazio@gmail.com`

October 2016

Abstract. We analyze global export data within the Economic Complexity framework. We couple the new economic dimension Complexity, which captures how sophisticated products are, with an index called logPRODY, a weighted average of the Gross Domestic Products per capita of a product's exporters. Products' aggregate motion is treated as a 2-dimensional dynamical system in the Complexity-logPRODY plane (CLP). We assign an average value of competition on the markets to points of the CLP, using the Herfindahl index. The motion of the products on the CLP consists in a fast movement away from zones where the competition is low, towards what we call the asymptotic zone, where competition is maximum. logPRODY of products in this area of maximum competition depends on their Complexity value. Since logPRODY is a proxy for the underlying set of countries that export a given product, displacements on the plane correspond to shifts in the export market. The observed dynamics can be modeled with an equation linking average speed to competition. The asymptotic logPRODY value corresponds to a market configuration that maximizes competition. We characterize it and call it asymptotic market; we find that its shape depends on the Complexity value of the product.

Abstract. We discuss here further information in support of the claims made by the paper.

Checking the validity of the $\vec{v} - \vec{\nabla}_k H$ relation

Here we report a more detailed comparison between the velocity field $\vec{v}$ and the gradient of the H field mentioned in the paper, calculated on the Feenstra dataset. We repeat the defining equation:

$$\vec{v} \simeq -k_x \frac{\partial H}{\partial x} \vec{x} - k_y \frac{\partial H}{\partial y} \vec{y} \equiv -\vec{\nabla}_k H. \tag{1}$$

We run a regression between horizontal and vertical components, moduli and directions of both $\vec{v}$ and $-\vec{\nabla}_k H$: the results are shown in figure S1 Fig. All the regressions are linear, with the zero-order coefficient set to be zero. The only exception is the regression of velocities along the vertical axis that, in all datasets, shows a clean linear trend and a few big outliers. It can be clearly seen by running a bootstrap on the regression, randomly taking away some of the points: in figure S2 Fig we show that the resulting Pearson's R^2 distribution is bimodal. This situation is caused by the very high velocities in the bottom right part of the $RCLP$ plane not matched by the model, because the actual maximum of the H field is slightly away from the corner, causing a very small gradient to appear in that area. To separate the outliers, we use a RANSAC regression [1]. We also show in figure S3 Fig that the equation holds for different resolutions of the grid used to divide the $RCLP$ plane, and if we calculate the $\vec{v}$ field by averaging displacements over an interval Δt bigger than one year.

BACI dataset results

We report here the results for the second dataset; both a representation of the $\vec{v}$ and $-\vec{\nabla}_k H$ fields in figure S4 Fig, the comparison via regression, in figure S6 Fig, and the market shape representation in figure S5 Fig, as done for the Feenstra dataset. The results are very similar across the two datasets, which span different time intervals, countries and products. This further confirms the consistency of our analysis. We also show a plot of all the individual points of the BACI dataset in figure S7 Fig.

Checking for size effects

There is a significant size effect on the value of the average Herfindahl index per box. The less points there are in a box, the higher is the variance on the average value, so higher values of the average are more likely. This has consequences on the meaningfulness of the H field, as its spatial pattern could conceivably be a mere consequence of the density pattern on the plane. To check for this effect, we build a null model in which each product is assigned a random value of the Herfindahl index chosen from all the observed values in the dataset. Careful examination of this model allows us to conclude that the spatial pattern of the observed H field is highly unlikely to arise from the size effect alone, see fig.S8 Fig, panel a). We also check Eq.1 against the fields obtained with the null model (which we will call $\tilde{H}$). We obtain a comparable significance level

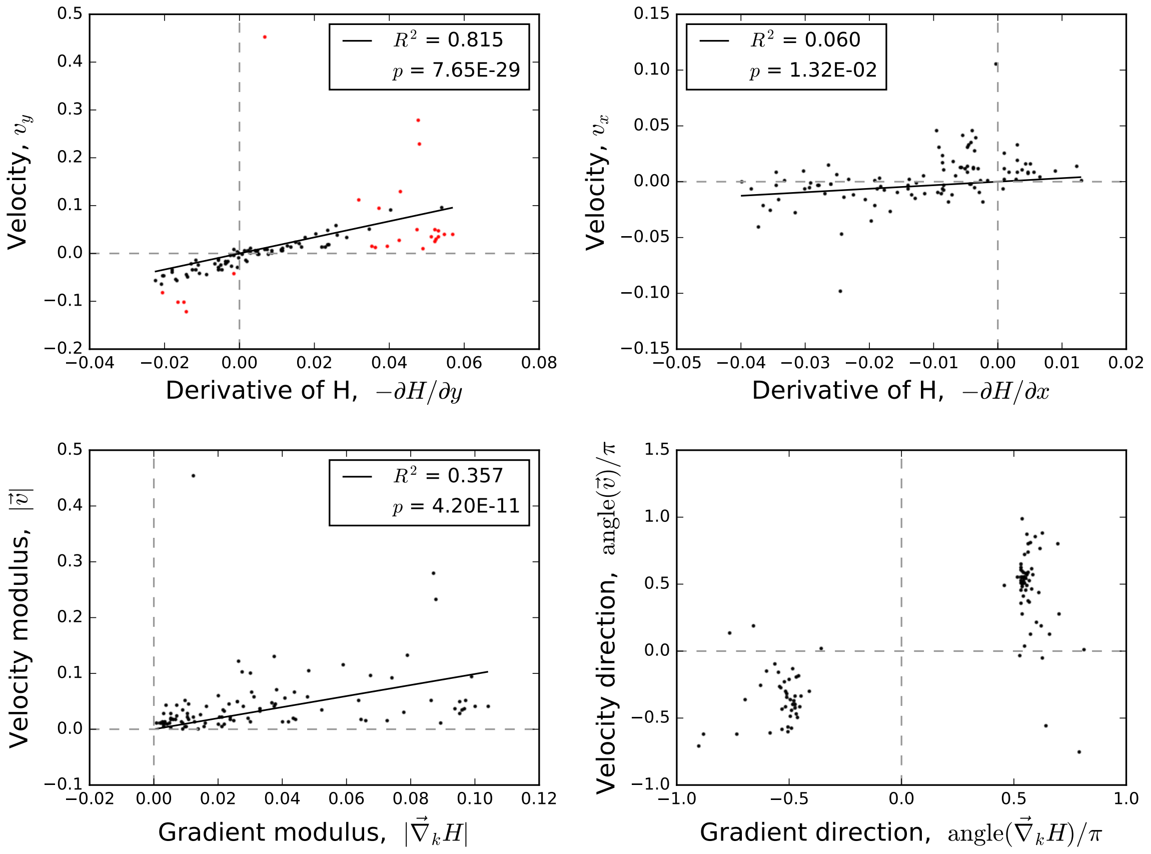

S1 Fig. Comparison between the 1-year average velocity field, $\vec{v}$, and the field obtained from the gradient of H defined in Eq.1. Calculations done on the Feenstra dataset. *Top:* comparison between the horizontal and vertical components of the field. In the left panel, showing the regression for velocities along the logPRODY axis, the points removed by the RANSAC regression are shown in red. The *Bottom:* Comparison between orientations and moduli of the field.

for the equation in about 1.5% of the cases, see Fig.S8 Fig, panel b). The results are similar on the BACI dataset. Given the combined two tests onto two different datasets, we conclude that the size effect is not originating the observed pattern of the H field.

Other definitions of the H field

We find that much better results can be obtained by calculating the Herfindahl index on the distribution of logPRODY weighs $\tilde{R}_{cp}$ in place of the market share distribution s_{cp}, as it reproduces the $\vec{v}_y$ component of the velocity field with significantly greater accuracy. This was expected, since $\tilde{R}$ enters directly in the definition of logPRODY.

As mentioned in the paper, similar results can be obtained by calculating the H

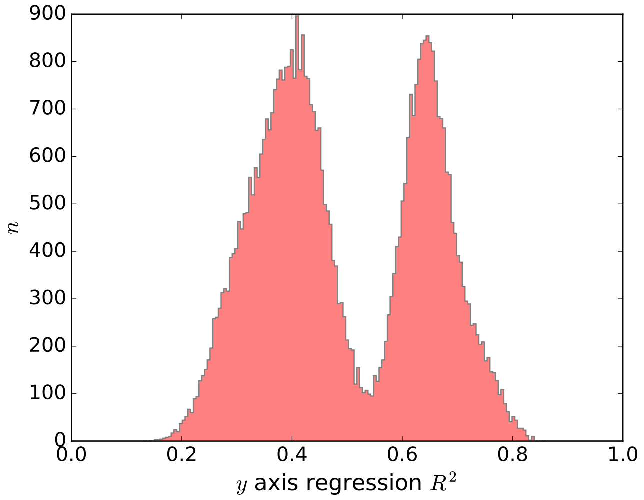

S2 Fig. Bootstrapping the regression of $\vec{v}_y$ versus $-\vec{\nabla}_k H$ at 1 year displacement time and 10x10 grid. Each iteration of the bootstrap randomly removed 10% of the samples, for 50k iterations. Two peaks are clearly visible: one at about $R^2 = 4$, and one at about .7. The bimodal distribution confirms our hypothesis that the points follow a very clear linear trend, with a few very big outliers. The peak at .7 is caused by the bootstrap randomly removing the outliers from the regression. The peak at .4 comes from the bootstrap removing points following the linear trend, and leaving the outliers in. This clear bimodal distribution allows us to use RANSAC, which is an algorithm apt to take out a few big outliers from a regression [1].

field with the $\tilde{R}$ values, i.e.

$$H_p^R = \sum_c \left(\tilde{R}_{cp}\right)^2; \qquad \tilde{R}_{cp} = \sum_c \frac{R_{cp}}{\sum_j R_{jp}} \tag{2}$$

Results are shown in figure S10 Fig, and the gradient obtained with $-\vec{\nabla}_k H^R$ is shown in figure S9 Fig.

Similar results can be obtained as well by substituting the Herfindahl index with the entropy of the distribution of market shares, and inverting the sign of the gradient:

$$S_p = \sum_c -s_{cp} \log(s_{cp}); \qquad \vec{v} = +k_x \frac{\partial S}{\partial x}\vec{x} + k_y \frac{\partial S}{\partial y}\vec{y} = \vec{\nabla}_k S \tag{3}$$

This was anticipated, and intuitively makes sense, as the Herfindahl index essentially measures the concentration of the discrete distribution of market shares, s_{cp}; therefore it anti-correlates very strongly with the entropy of such distribution. For the values of the S and H field, a linear relation is a good enough fit, as shown in figure S11 Fig, even though we find that the actual relation is not linear, as discussed further on. The test of Eq. 3 is shown in figure S12 Fig, while the field obtained is in figure S11 Fig.

In figure S12 Fig we show the results of the analysis if one substitutes H with S:

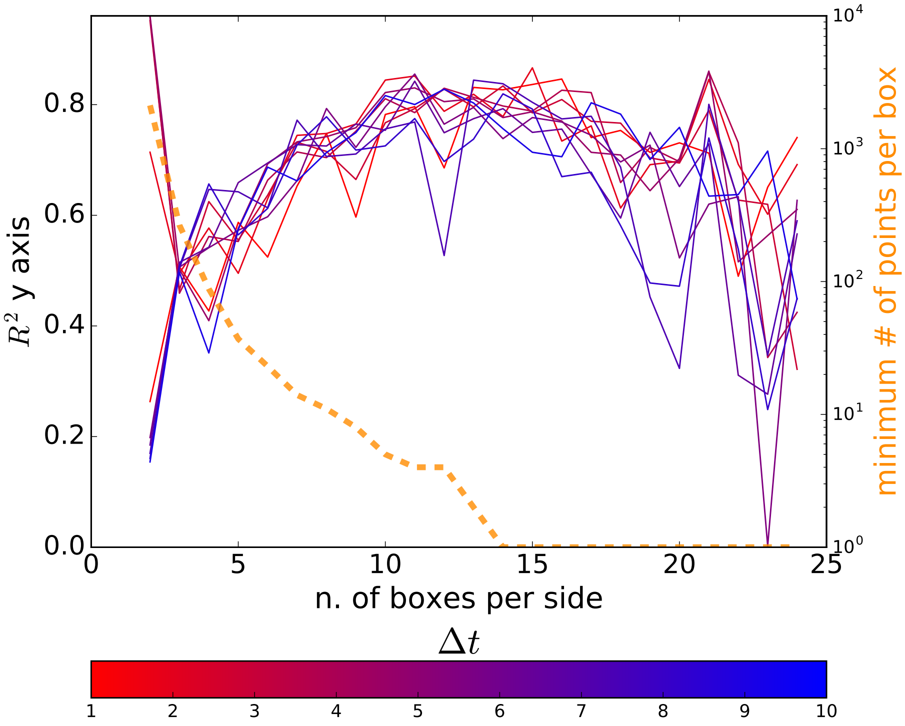

S3 Fig. A plot of the R^2 values obtained by regressing $\vec{v}_y$ versus $-\vec{\nabla}_k H$ at various resolution and displacement time values, Feenstra dataset. On the horizontal axis there is the resolution of the grid, with the number of boxes per side. In blue to red color, we show the time interval used to calculate the products' displacements, which are then averaged into $\vec{v}$. The yellow line indicates the minimum number of points per box at a given resolution. The accuracy of our model's prediction peaks at a resolution between 10 and 15. Less resolution is probably too little detail to capture the features of the system. Accuracy starts to drop, especially for longer time displacements, as soon as the number of points per box is 1; this is probably caused by an increase in noise in the less populated areas of the plane. All the regressions are linear, calculated with RANSAC, and with the zero-order coefficient set to zero by hypothesis.

1. Ubiquity

One possible critique of the model exposed in the paper is that the Herfindahl index should be inversely correlated to Ubiquity, defined as:

$$U_p = \sum_c M_{cp}, \tag{4}$$

where M_{cp} is the adjacency matrix of the bipartite country-product network. Ubiquity measures the number of countries that export a given product. The field obtained by regressing average Ubiquity per cell, though, is quite different from the one obtained

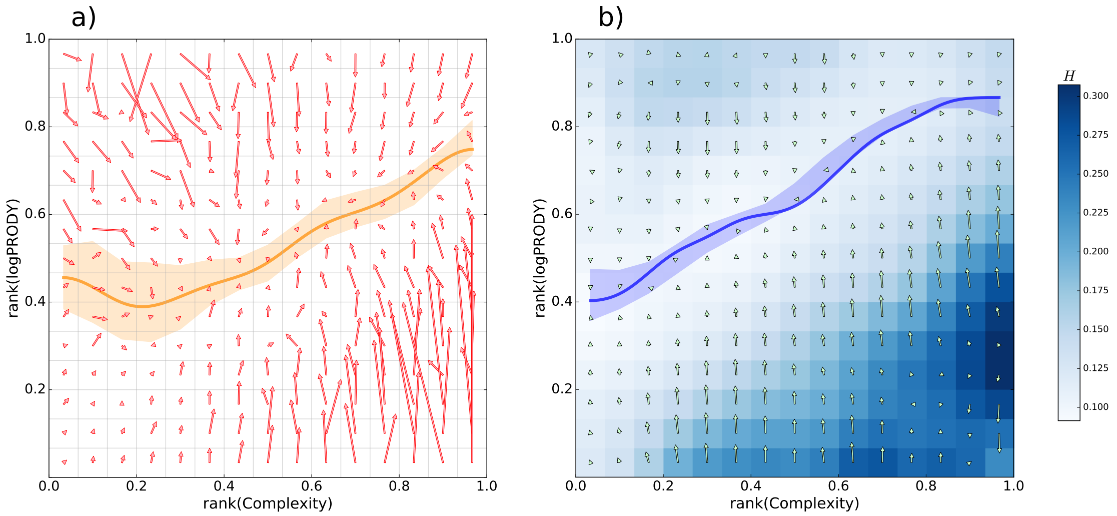

S4 Fig. $\vec{v}$ and $-\vec{\nabla}_k H$ fields for the BACI dataset. The results are extremely similar to those shown in the paper for the Feenstra dataset, with great consistency across datasets. **Panel a).** In red, the average velocity field of the products on the *RCLP* plane, $\vec{v}$. The asymptotic zone is marked by the orange line, together with the bootstrap result for the 5% confidence interval. **Panel b).** Average Herfindahl per box, the H field (in blue), with the derivatives calculated according to eq. 1 (green arrows). The line is the kernel regression of the minima of the Herfindahl field per column, with 5% confidence interval on the value superimposed. All the arrows in both panels are to 1:1 scale with each other and with the plot's axes.

by regressing the Herfindahl field, as can be seen in Fig.S13 Fig. This is because the average Ubiquity of products decreases with increasing Complexity; therefore the left part of the *RCLP* plane has higher Ubiquity values than the right. For this reason, Eq.1 does not give good results.

Stationarity of the points distribution on the RCLP plane

To support our claim that the distribution of the points on the RCLP plane is stationary, even though the velocity field $\vec{v}$ seems highly non-stationary, we remind that $\vec{v}$ only measures the average velocity of points going out of each box. The displacement is counted in a box's average if the product moved out of the box in the subsequent timestep. $\vec{v}$ does not show how products enter the boxes. We show this "inward" velocity $\vec{w}$ (i.e. the average velocity of points entering each box) in **??**. The vectors are not to scale with the ones showed in the paper, so in order to allow comparison, we show both $\vec{w}$ and $\vec{v}$ represented with the same scale in figure S15 Fig. We also show the total fluxes in and out of each box in figure S17 Fig, as well as their integral, the total occupation numbers timeseries for each box in figure S16 Fig.

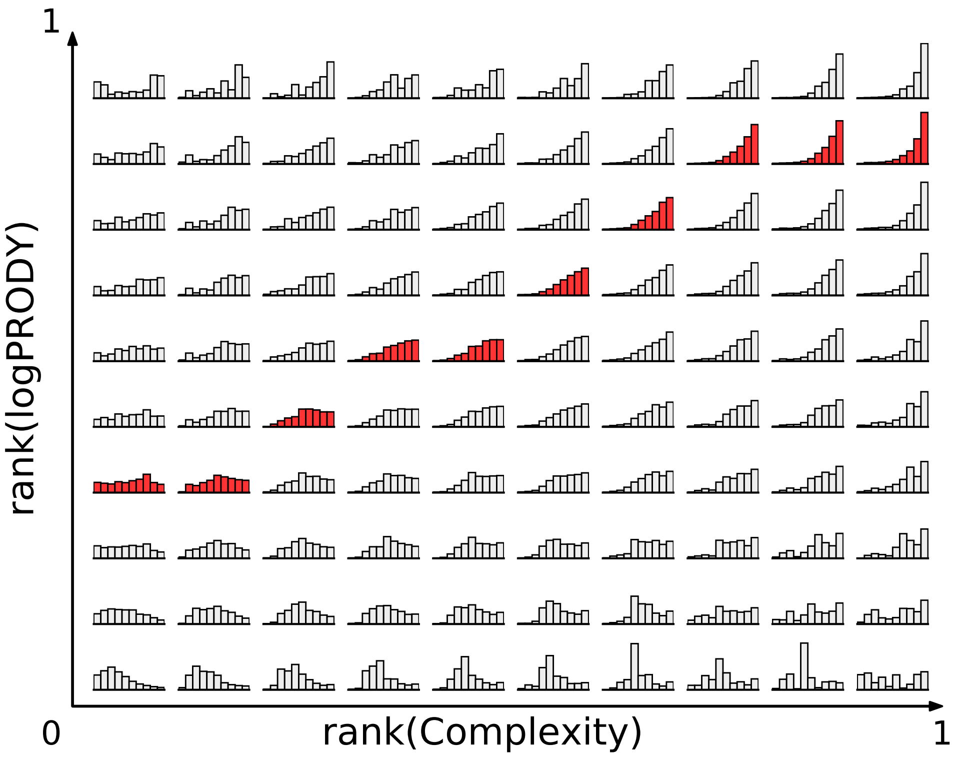

S5 Fig. Average RCA weights per box, BACI dataset. For each box b we plot a histogram showing the average $\tilde{R}$ values of countries exporting the products contained in b. Each bar of the histograms shows the average RCA of countries with a fitness value between two consecutive deciles of the fitness distribution. We remind that $\tilde{R}$ represents the share of a product in a country's total exports. Here we see the same patterns found in the Feenstra dataset: the distributions on the minima of H going from flat for the lowest Complexity level to markedly peaked on high fitness for the highest Complexity. Again, the results show consistency.

S-H relationship

We find that there is a particular relationship between the Herfindahl value of a distribution and its corresponding entropy value. In figure S18 Fig we show a scatter plot of the Herfindahl index vs. the entropy value for individual products' market share distributions. It actually consists of the relation between the expected values $\int xf(x)dx$ and $\int \log(x)f(x)dx$, where the $f(x)$'s are the empirical distributions of market shares.

References

[1] Martin A. Fischler and Robert C. Bolles. Random sample consensus: a paradigm for model fitting with applications to image analysis and automated cartography. *Communications of the ACM*, 24(6):381395, jun 1981.

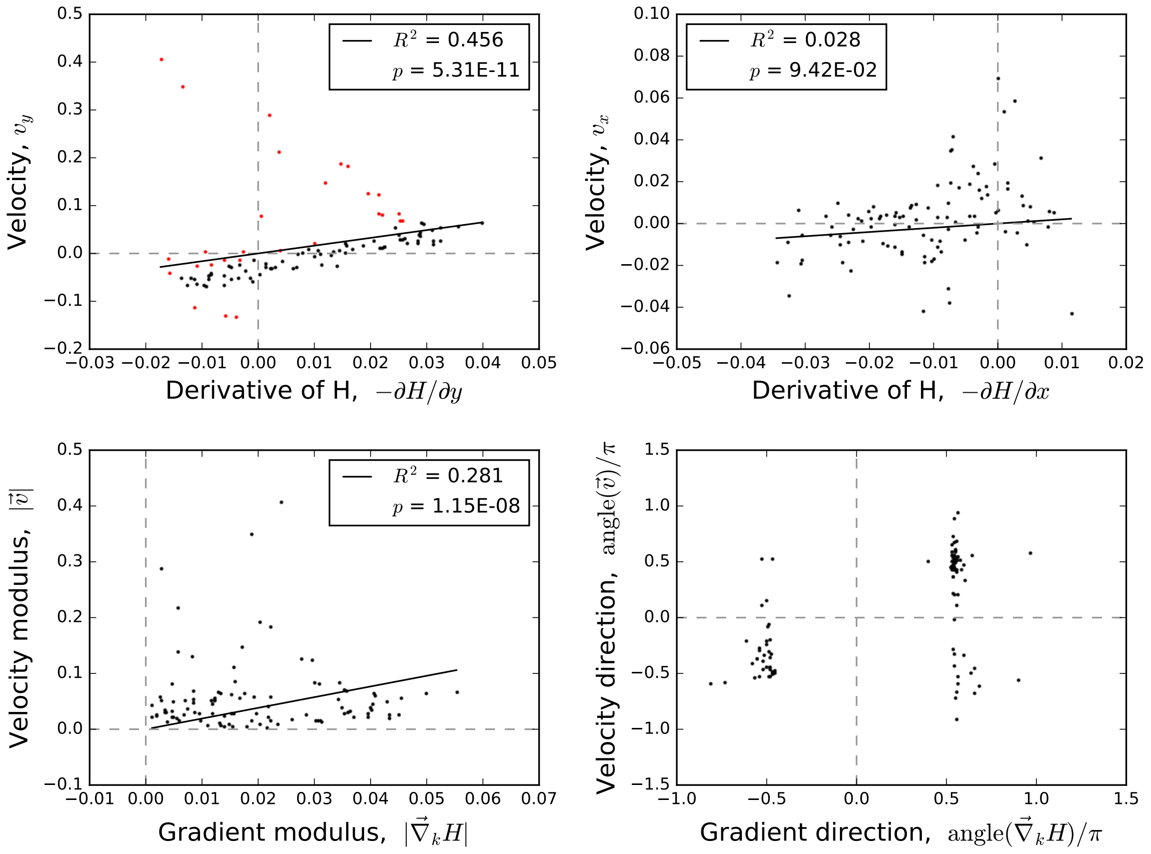

S6 Fig. Comparison between the 1-year average velocity field, $\vec{v}$, and the field obtained from the gradient of H defined in the paper. Calculations done on the BACI dataset. *Top:* comparison between the horizontal and vertical components of the field, the latter regressed with RANSAC. *Bottom:* Comparison between orientations and moduli of the field.

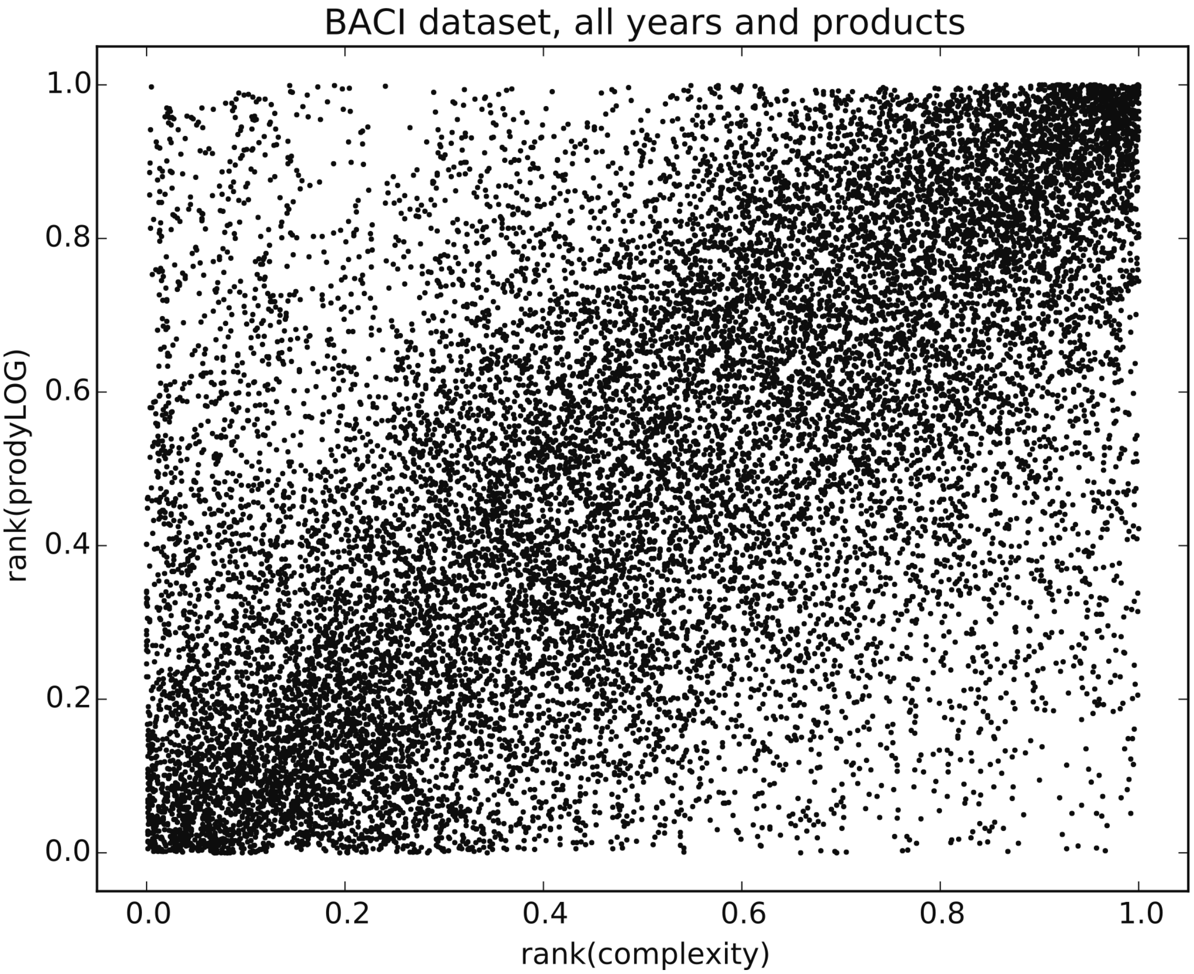

S7 Fig. All individual data points of the BACI dataset. One can clearly see higher density on the diagonal, and a decreasing number of points in the upper left and lower right quadrants of the RCLP plane.

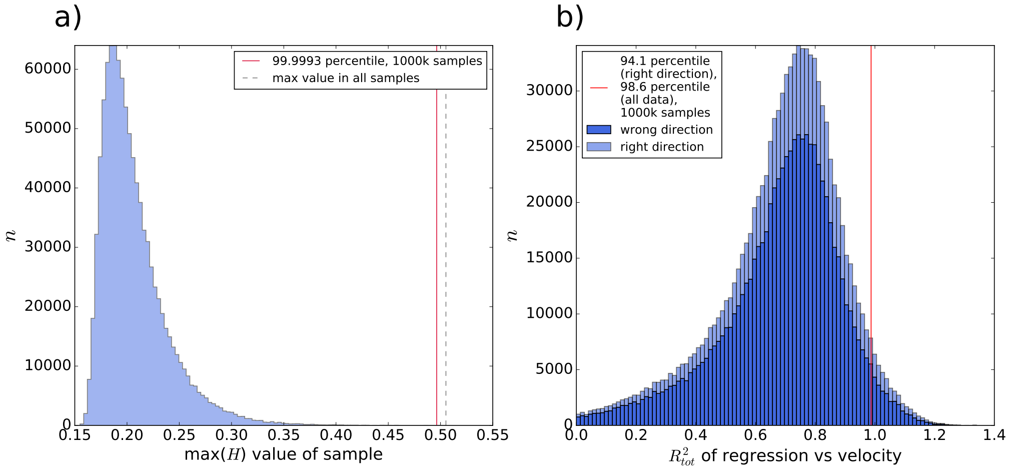

S8 Fig. Panel a) Maximum value of the H field. Given that the less points one has per box, the higher the standard deviation for H in that box is, one expects to find higher values of H where the density of points is lowest on the $RCLP$ plane. We check whether the null model can produce a $\tilde{H}$ field with the same highest value found in H, which we will call $\max H$. Running the model 1M times produced few samples of comparable highest value $\max \tilde{H}$. In the histogram we show the frequency of $\max \tilde{H}$; the vertical line shows $\max H$ for the Feenstra dataset. A similar result (not shown) is found in the BACI dataset. **Panel b)** Check of Eq.1's validity versus the null model. We ran the null model 1M times, and for each $\tilde{H}$ field obtained we calculated the correlation between $\tilde{H}$'s and $\vec{v}$'s vertical and horizontal components with a linear regression. The sum of the two Pearson's R^2 coefficients obtained from this process, R^2_{tot} (which is a number between 0 and 2), is used here as a measure of significance for the $\tilde{H}$. In this histogram we show the frequency of the R^2_{tot} values; the vertical line represents R^2_{tot} obtained from the H field. The null model produces gradients that correlate both positively or negatively with each component of $\vec{v}$: in some cases, one gets a high R^2_{tot}, but the field being reproduced is the inverse of $\vec{v}$. In light blue, we show the fraction of the $\tilde{H}$'s that correctly reproduce the direction of $\vec{v}$ on both components, while dark blue represents the fraction that reproduces at least one of the components of $-\vec{v}$. The result is that, among all generated $\tilde{H}$ fields (both with right and wrong direction), those with a significance level higher than that of H are 1.5%.

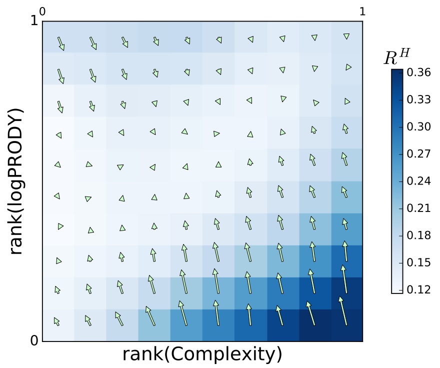

S9 Fig. The field obtained by calculating the gradient of the H^R field, defined in equation 2

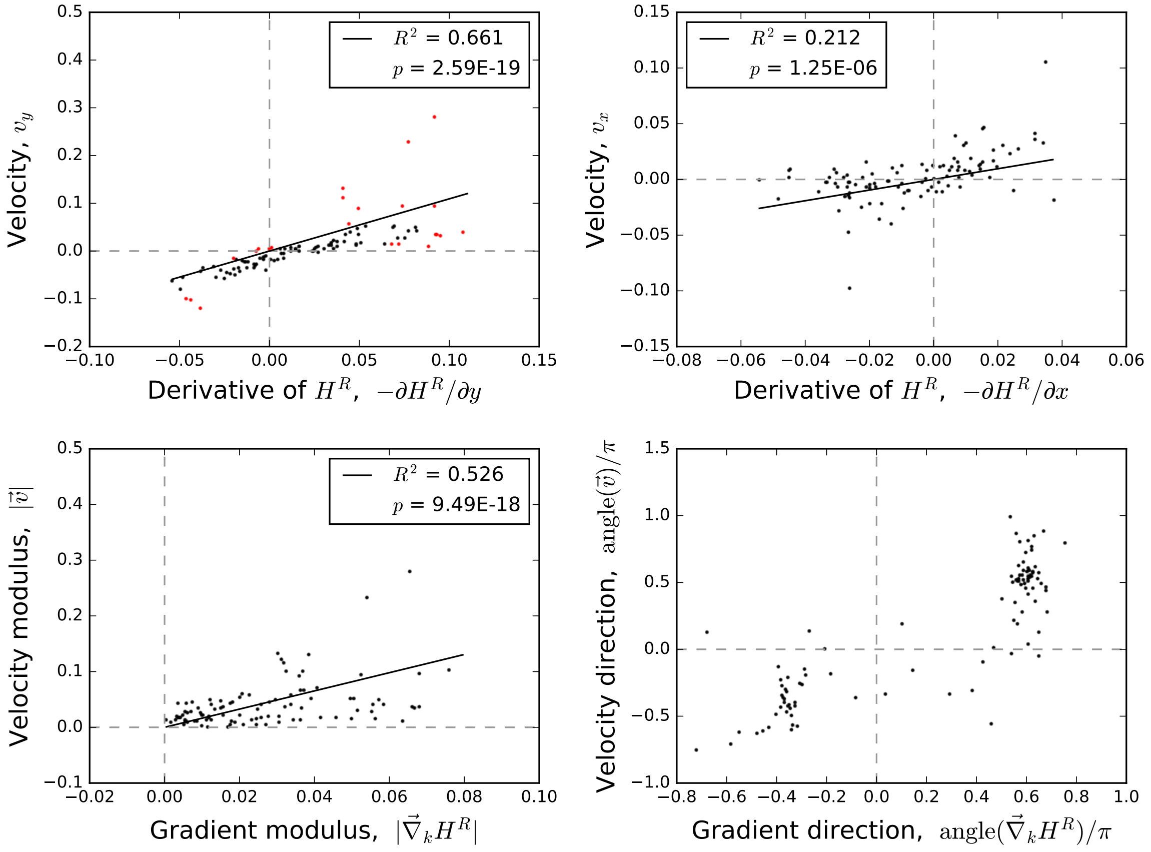

S10 Fig. Comparison between the 1-year average velocity field, $\vec{v}$, and the field obtained from the gradient of H^R defined in equation 2. Calculations done on the Feenstra dataset. *Top:* comparison between the horizontal and vertical components of the field *Bottom:* Comparison between orientations and moduli of the field.

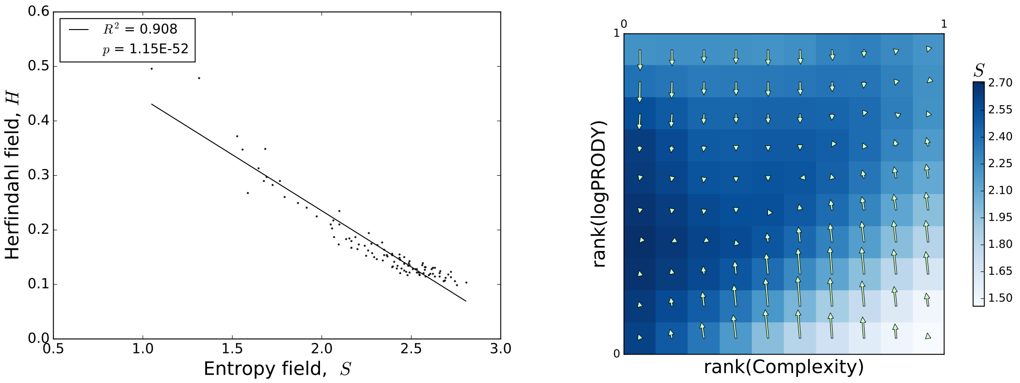

S11 Fig. *Left:* Relation between values of the S and H field in the BACI dataset. Left, in black, a linear fit, which is good enough for the fields, that aggregate many individual points. The actual relationship between entropy and Herfindahl index of a distribution is not linear, as we show in the next section. *Right:* The field obtained by applying equation 3

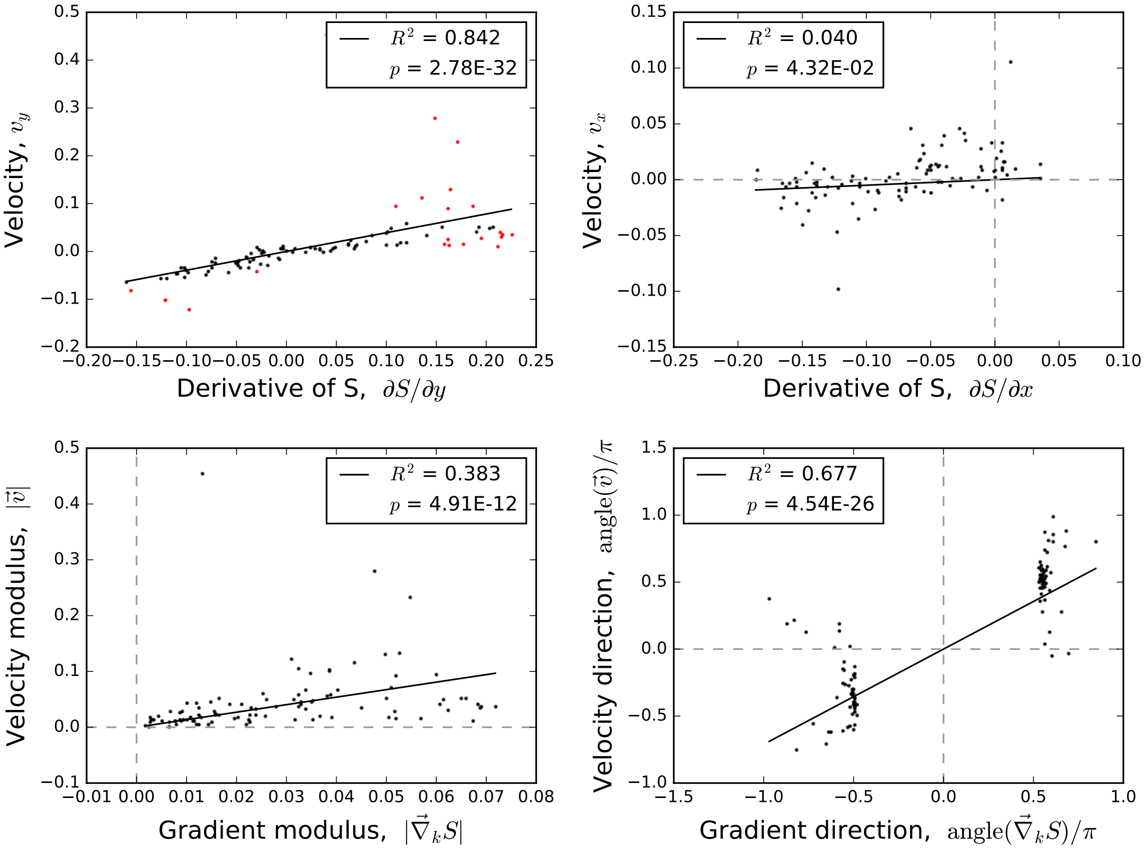

S12 Fig. Comparison between the 1-year average velocity field, $\vec{v}$, and the field obtained from the gradient of S. Calculations done on the Feenstra dataset. *Top:* comparison between the horizontal and vertical components of the field *Bottom:* Comparison between orientations and moduli of the field.

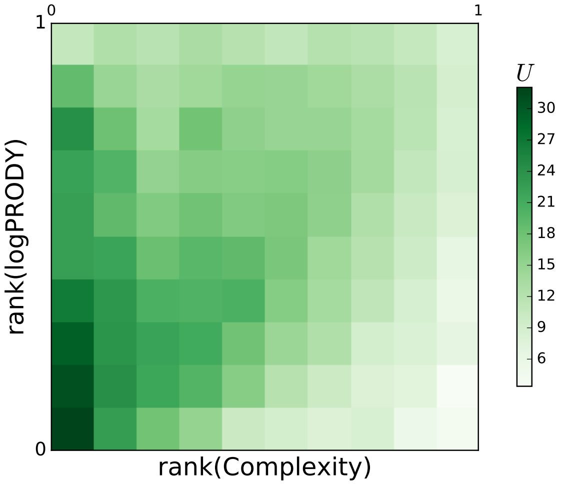

S13 Fig. In green, the field obtained by regressing the average value of U (Ubiquity) per box in the $RCLP$ plane. It is different from the field obtained by regressing H, because the average Ubiquity of products decreases with increasing Complexity; therefore the left part of the $RCLP$ plane has higher values than the right. The plot shows the regression for the Feenstra dataset.

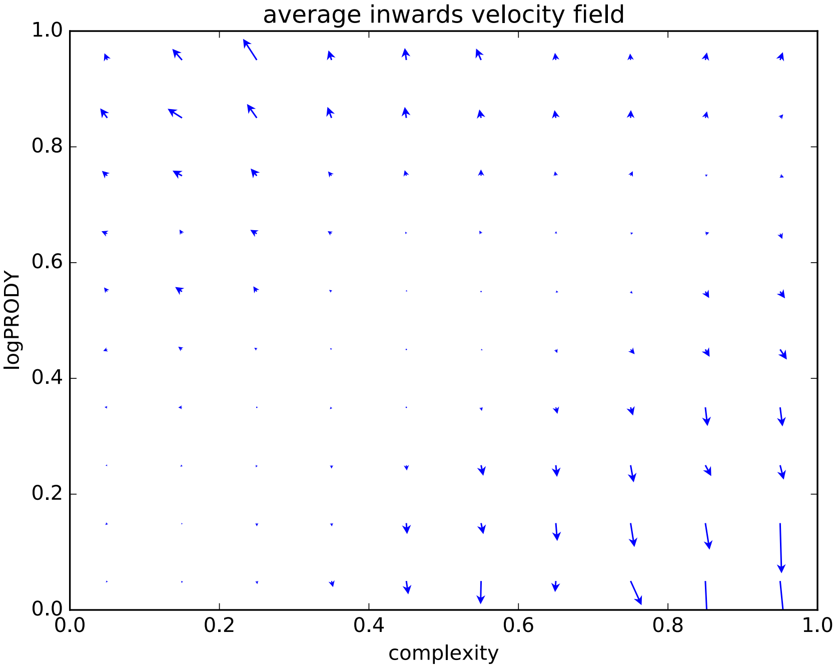

S14 Fig. "inward" velocity field $\vec{w}$. To calculate this field, considered all products that entered a given box, and averaged their displacements. The vectors are not to scale with those shown in the rest of the paper. To allow comparison, we present a depiction of both $\vec{w}$ and $\vec{v}$ fields in figure S15 Fig. The figure refers to the BACI dataset.

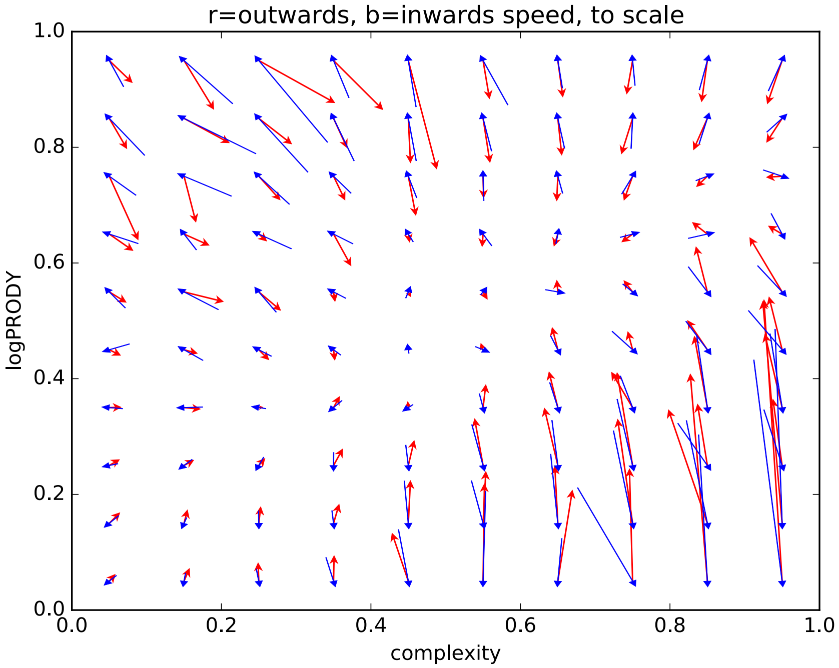

S15 Fig. Both $\vec{w}$ and $\vec{v}$ fields, to scale with each other. The figure refers to the BACI dataset.

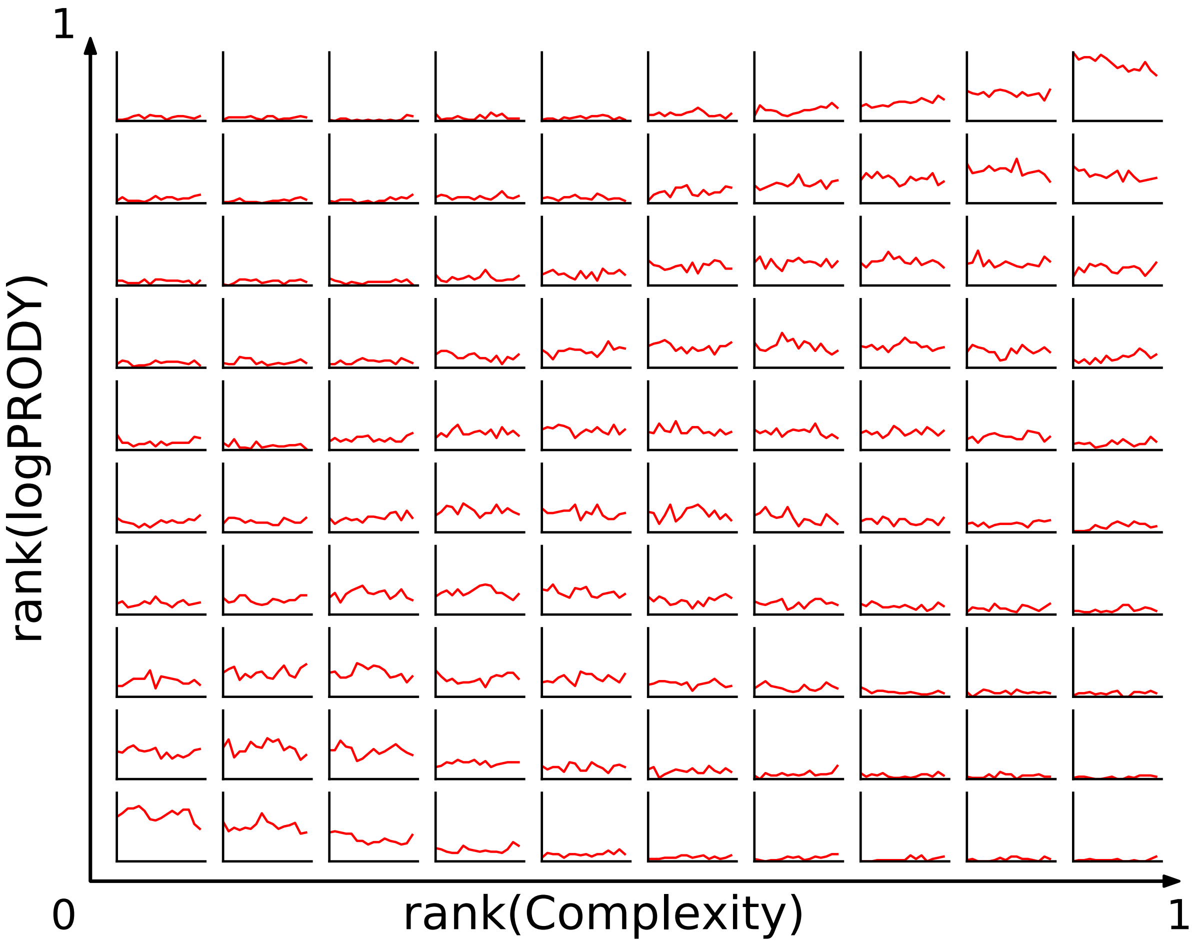

S16 Fig. Occupation number timeseries for each box onb the RCLP plane. Each of the plots in this figure shows the evolution in the number of products contained in each box. The horizontal axis of the plots represents time in years, and the vertical axis the number of points. The plots are to scale relative to each other. The figure refers to the BACI dataset.

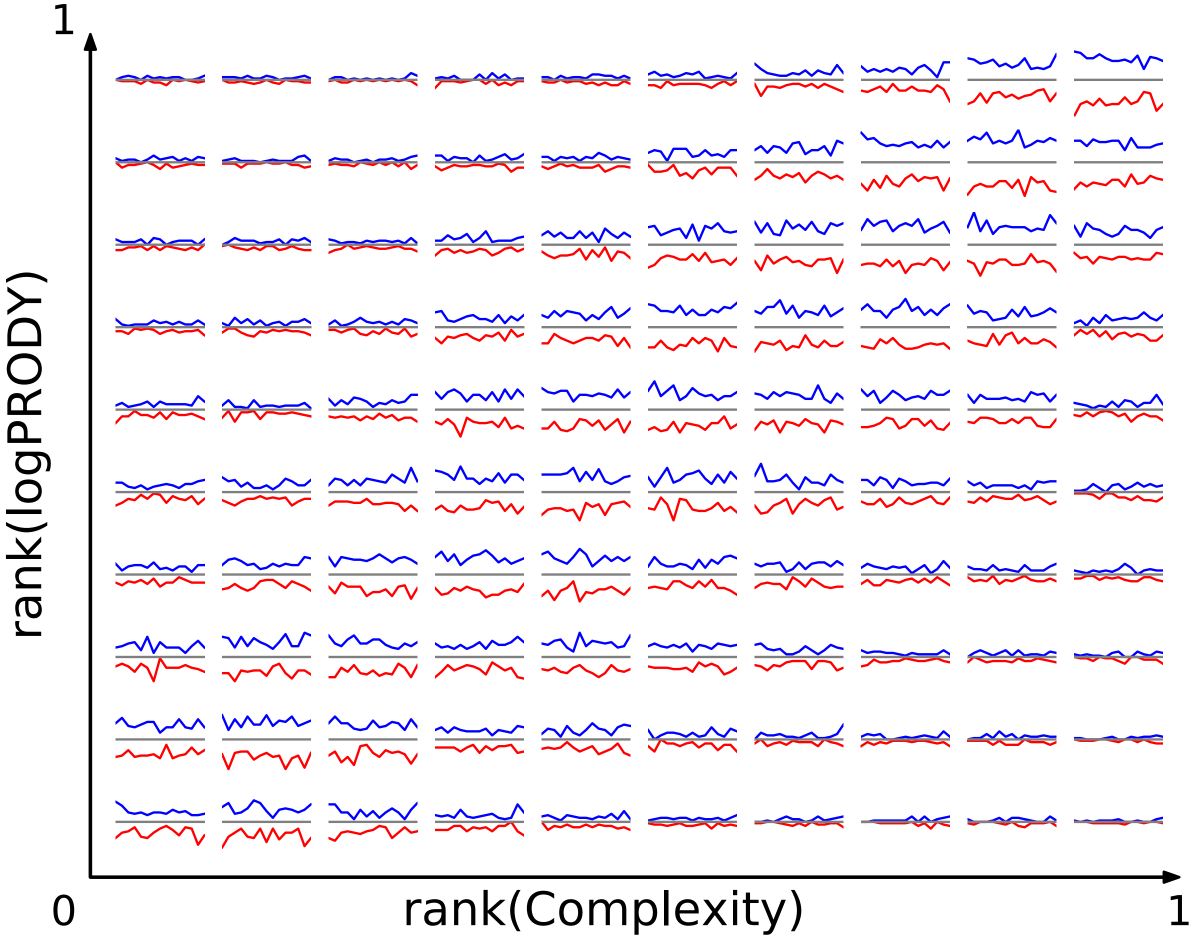

S17 Fig. Inward (blue) and outward (red) fluxes for each box on the RCLP plane. Each of the plots in this figure shows the yearly change in the number of products contained in each box. The horizontal axis of the plots represents time in years, and the vertical axis the difference in number of points (for the outward flux the difference is negative). The plots are to scale relative to each other. The figure refers to the BACI dataset.

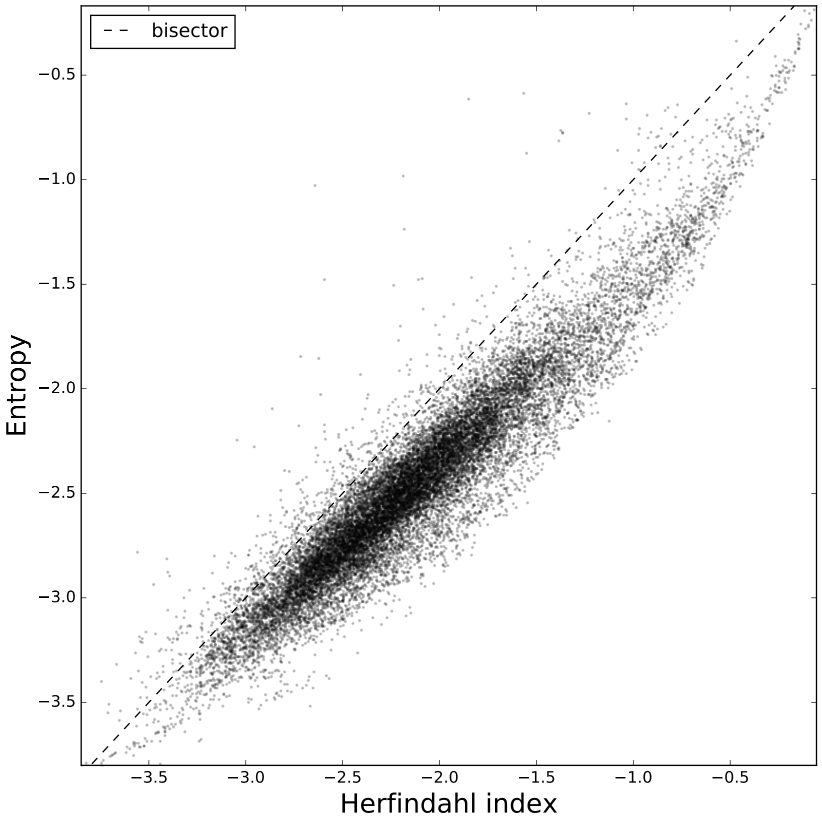

S18 Fig. A scatter-plot of the Herfindahl index vs. the entropy value for individual products' market share distributions, from the BACI dataset.
